# Mass Isotopologue Distribution of dimer ion adducts of intracellular metabolites for potential applications in ^13^C Metabolic Flux Analysis

**DOI:** 10.1371/journal.pone.0220412

**Published:** 2019-08-21

**Authors:** Charulata B. Prasannan, Vivek Mishra, Damini Jaiswal, Pramod P. Wangikar

**Affiliations:** 1 Department of Chemical Engineering, Indian Institute of Technology Bombay, Powai, Mumbai, India; 2 DBT-Pan IIT Center for Bioenergy, Indian Institute of Technology Bombay, Powai, Mumbai, India; 3 Wadhwani Research Center for Bioengineering, Indian Institute of Technology Bombay, Powai, Mumbai, India; Universitat Bremen, GERMANY

## Abstract

^13^C Metabolic Flux Analysis (^13^C-MFA) is a powerful tool for quantification of carbon flux distribution in metabolic pathways. However, the requirement to obtain accurate labeling patterns, especially for compounds with low abundance, poses a challenge. Chromatographic separation and high sensitivity of the modern mass spectrometers (MS) alleviate this problem to a certain extent. However, the presence of derivatives such as in-source fragments, multimer ion adducts, and multiply charged ions result in reduced intensity of the molecular ion. While multimer ion adducts have been reported in the field of metabolomics, their presence is considered undesirable in quantitative studies. Here, we demonstrate a novel application of dimer ion adducts in calculating the mass isotopologue distribution (MIDs) of the corresponding monomer ions for public domain and in-house generated datasets comprising of ^13^C-labeling time-course experiments. Out of the 100 standard compounds analyzed, we could detect multimer ion adducts in 24 of the intermediate metabolites. Further, a subset of these multimer ions were detected in all the biological samples analyzed. Majority of these ion adducts were either not detected in the original study or labeled as a putative features. Regression analysis was performed to estimate the monomer MIDs from those of the dimer. This resulted in accurate estimation regardless of the biological system, chromatographic method, the MS hardware, or the relative abundance of the dimer ion. We argue that this analysis may be useful in cases where satisfactory data cannot be extracted from the chromatographic peaks of the monomer ions.

## Introduction

^13^C Metabolic flux analysis (^13^C-MFA), which has been in development since the 1990s, is an important tool for the study of metabolism [1] that uses the^13^C labeling patterns of key metabolites and a metabolic network model of the organism to estimate the carbon flux distribution. The conventional stationary (ST) approach uses the mass isotopologue distributions (MIDs) of proteogenic amino acids while the more recently developed non-stationary (INST) MFA uses the labeling pattern of intracellular metabolites for quantitative estimation of fluxes [2–8]. INST-MFA has become an indispensable tool for systems that are not amenable to steady state ^13^C MFA experiments [9–11]. These include photoautotrophic and methylotrophic organisms that utilize single carbon substrates such as carbon dioxide or methane and thus attain uniform labeling at isotopic steady state conditions [12]. Furthermore, concentrations of intracellular metabolites are low and therefore tracking the label distribution is a challenge.

With the advent of high resolution mass spectrometers (HR-MS), efficient extraction methods specific for the strain, novel data acquisition workflows, and numerous data analysis software, it is possible to accurately quantitate the mass isotopologue distribution (MIDs) of a number of metabolites [13–16]. The list of metabolites still needs to expand to include metabolites that are further away from the central carbon pathway and include non canonical metabolites. This may require a) thorough analysis of the collected data with respect to the peaks present and b) improvement in both LC and MS methods to detect various classes of compounds. Due to the nature of LCMS data, each metabolite is represented by multiple peaks, which comprise of ion adducts (singly or multiply charged), isotopes, multimer ion adducts, and in-source fragments [17]. Several approaches/software tools make use of this information with the exact mass match to reduce the candidate numbers for metabolite identification [17].

Both small molecules and large biomolecules can form multimer complexes (dimers, trimers, and tetramers) [17]. The detection of these complexes has increased with the emergence of soft ionization techniques such as electrospray, which has the ability to desorb intact and multiply charged high molecular weight ions from the aqueous to the gaseous phase [18]. The abundance of the multimer ions is lower than that of the monomer ions in most cases, however, it can vary depending on the collisional activation parameters and curtain gas temperature used in the parameter settings [19,20]. Further, strong concentration dependence of the abundance of multimer ions has been reported for certain antimalarial drugs [20]. However, to the best of the authors’ knowledge, there are no reports that provide an exhaustive list of metabolites for which multimers are detected.

In the negative ion mode, the most frequent adducts formed are in the form of [M-H]^-^, [M-H-H_2_O]^-^ and [2M-H]^-^ ions [21]. Apart from a few reports that use in-source fragments for identification and quantification of the absolute concentration of metabolites [22,23], the use of adducts has been prevalent for compound identification. One possible reason is that the information about these types of adducts are not readily available in databases and therefore they are not annotated readily. Further, they distribute the metabolite pool into various m/z features thereby resulting in a compromised monomer peak and non-linear calibration curves [20]. This is true in many cases especially when the concentration of the compounds of interest is low. In this study, we describe a direct method for the use of multimer adducts (dimers, and trimers) detected in the samples from various biological system, towards the ^13^C-MFA studies. The phenomenon of adducts formation is utilized to obtain a measurement for monomer MID from dimer or trimer MID. This is first ever report for such an application and it will be useful in many cases where the direct quantification of monomer MIDs is compromised. We also identify multimer ion adducts, [2M-H]^-^ and [3M-H]^-^ for various standard compounds and report their fragmentation pattern towards their addition into relevant databases.

## Experimental section

### Materials

Individual metabolite standards (IROA-MSMLS) and other standard chemicals of the highest grade available were purchased from Sigma-Aldrich (Saint Louis, MO). The tributylamine (puriss. plus, ≥ 99.5%), acetic acid (ACS reagent, ≥ 99.7%) and other reagents for LCMS analysis were also purchased from Sigma-Aldrich (Saint Louis, MO).

### Datasets used in this study

Data from time course measurements for label incorporation using ^13^C substrates were used. Two published datasets from the reported studies on *B*. *methanolicus* MGA3 [13] (henceforth referred to as MGA3), and human stem cell derived cultured reticulocytes [24] (henceforth referred to as CD34+), and one in-house dataset on a cyanobacterial strain, *Synechococcus* sp. PCC 7002 (henceforth referred to as PCC 7002), were analyzed for multimer adducts. The data collected on the strain PCC 7002 has been submitted to Metabolomics Workbench (http://dx.doi.org/10.21228/M87384). The in-house time course measurement (unpublished results) on strain *Synechococcus elongatus* PCC 11801 (henceforth referred to as PCC 11801) [25] was shown to highlight the omnipresence of these multimer ion adducts. The data on MGA3 and CD34+ was obtained from Metabolights (study identifier number MTBLS228) and Metabolomics Workbench (http://dx.doi.org/10.21228/M8002N) repositories, respectively. The details of the experiments are mentioned in the original studies [13,24]. Briefly, the MGA3 strain was grown in bioreactors and metabolic labeling measurements were performed using ^13^C methanol. Time course labeling data on metabolites were analyzed using the LC-HRMS using nanoscale ion-pair RP-HPLC method with LTQ-Orbitrap instrument in negative ion mode.

The labeling in CD24+ cells was performed using 50% U-^13^C-glucose and samples were collected 1 hour and 20 hours after the carbon source switch. Samples were analyzed by HILIC chromatography coupled to a Q Exactive.

### Labeling and extraction

The cyanobacterial strain, PCC 7002 was cultured in bioreactors with ASNIII + BG11 medium at 38°C illuminated with external fluorescent lights (250 μEm^-2^S^-1^) [8]. A ^13^C-Sodium bicarbonate solution was added to an exponentially growing culture and time-course samples were collected. The culture was filtered quickly in the presence of light before quenching with 100% cold methanol. Metabolites were extracted using methanol-chloroform-water mixture and the aqueous layer was dried using a vacuum centrifuge and stored at -80°C until it was used.

### Chromatographic method

Metabolites sample was analyzed using UHPLC (Shimadzu, Nexera LC-30 AD, Singapore) coupled with a Triple-TOF 5600 mass spectrophotometer (Sciex, Framingham, MA) with an electrospray ionization (ESI) source. A previously reported chromatographic method using a reverse phase column and ion paring reagent was used for the separation of metabolites [15]. Briefly, a 10 μL of sample was analyzed in a synergi hydro RP column using a gradient system of buffer A (tributylamine) and buffer B (methanol). The gradient profile used was t = 0 min, 0% B; t = 2 min, 0% B; t = 8 min, 35% B; t = 10.5 min, 35% B; t = 15.5 min, 90% B; t = 20.5 min, 90% B; t = 22 min, 0% B at a flow rate of 0.3 mL/min.

### Mass spectrometer parameters

The Triple-TOF 5600 mass analyzer was operated in the negative ion mode with an ion spray voltage of 4500V and interface heater temperature of 450°C. The ion source gas1 and gas 2 (GS1 and GS2) and curtain gas were set at 40, 40, and 35 respectively. The collision energy used were selected for each standard metabolite based on the m/z value of the precursor ion. A collision energy of -30 eV was used for the sample injections.

### Data processing

The extracted ion chromatograms (XIC) of precursor ions for monomer and dimer ions were analyzed using peak visualization software PeakView 2.2 (Sciex, Framingham, MA) and MasterView 1.0 (Sciex, Framingham, MA). All the datasets used were also analyzed using XCMS online [26–30] to identify metabolites and their multimer ion adducts. The mass isotopologue distribution (MIDs) of the metabolites were quantitated using DynaMet [13] for dataset on MGA3, and geoRge [21] for datasets CD34+, and PCC 7002/PCC 11801 respectively. The optimized parameters used for the analysis of these datasets are presented in S1 Table.

### Statistical analysis

Two replicate measurements were analyzed for each study. An unpaired parametric t-test (Welsh t-test) was implemented for detection of dimer ion adducts using XCMS online[26–30].

### Calculation of MIDs of monomer/dimer ions

Here we estimate the MIDs of multimer ions using the data from MIDs of monomer ions and vice versa. The possible combination of equations depends on the probability of multimer formation and the number of carbon atoms. For example, in a 3 carbon molecule, the possible monomer isotopologues are m+0, m+1, m+2, and m+3. For the dimer ion adduct of the same compound, the isotopologues formed will be M+0, M+1, M+2, M+3, M+4, M+5, and M+6. The possible combination of monomer units to form M+1 of the dimer are (m+0/m+1) and (m+1/m+0). Therefore, the probability of M+1 will be the product of each occurring event (m+0) *(m+1). Since the two combinations are mutually exclusive, the probability of M+1 of dimer will be sum of the two events, 2(m+0)(m+1). Similarly, the distribution of monomer units can be calculated from the dimer units. However, this will lead to a scenario with a greater number of equations and fewer unknowns, an over-determined system. We solve this overdetermined system by using least square analysis [31]. The least square analysis will provide a best fit solution to the over-determined system. This is best done using an optimization method such as ‘fsolve’ in MATLAB. In our fit we used a tolerance value of 1e-8 as stopping criteria for MATLAB iterations. The matlab code used to perform this analysis is provided as text file (S1 Text).

## Results and discussion

### Presence of multimer adducts in standard injections

As a first step, over 100 intracellular metabolite standards were analyzed using the LCMS method described in the methods section. Other than the information on retention time and fragmentation patterns, the analysis also provided details on the formation of multimer ion adducts. Table 1 shows the list of 24 metabolites, where multimer (dimer and trimer) adducts were detected. The overlay plots describing the peak overlap in terms of retention time and the fragmentation pattern of the monomer, dimer, and trimer (wherever detected) peaks are shown in the supplementary information (S1 Fig—S24 Fig). The collision energy used for each metabolite was selected based on the m/z of the monomer ions. Since the multimers formed were of larger m/z value, the collision energy chosen for the monomer was not sufficient to obtain all possible fragments observed from the dimer ion adducts. However, in the fragmentation pattern of each of the multimer ions, we could detect the corresponding monomer ion fragment and a subset of fragments from the mass spectrum of the monomer thereby confirming the monomer-multimer association. Note that although the monomer ion shows the highest relative intensity in the mass spectra of the dimer ions, the absolute intensity of the monomer in this MS2 data was not sufficient to quantify the MIDs of the monomer directly. The information on multimer ion adducts can be potentially integrated into the metabolite identification software for the annotations of multimer peaks. Under our MS data acquisition conditions, the trimer peak detected was always less intense than the monomer peak (refer to the supplementary figures). However, for a few metabolites such as sucrose, shikimate, phosphoenolpyruvate, N-acetyl glucosamine, and uridine the dimer peaks were more intense than the monomers (supplementary information). In case of 5-MethylthioAdenosine (S18 Fig), the dimer peak was approximately 10 fold more intense than the monomer peak.

**Table 1:** Multimer ion adducts identified in metabolite standards. Over one hundred pure metabolite standards were analyzed using the described LCMS method. Multimer ion adducts were identified in 24 of the metabolite standards.

Metabolite Name^a^	RT^b^ (min)	m/z		
		[M-H]^-^	[2M-H]^-^	[3M-H]^-^
N-Acetyl glucosamine	1.4	220.08	441.17	
Sucrose	1.6	341.1	683.2	
Uridine	6.2	243.06	487.13	
D-Glucono-1,5 Lactone	6.9	195.05	395.1	
Gluconic acid	7.3	195.05	391.11	
Shikimate	7.4	173.04	347.09	
Inosine	7.7	267.07	535.15	803.23
Quinate	7.8	191.05	383.11	
Dihydroorate	7.9	157.02	315.06	
Glucose 6 Phosphate	8.9	259.01	519.05	779.08
Tryptophan	9.4	203.08	407.17	
Ribose 5 Phosphate	9.7	229.01	459.03	689.05
Sedoheptulose-7-phosphate	9.9	289.01	579.07	869.12
Ribulose 5 Phosphate	10.1	229.01	459.03	689.05
Deoxy-Cytidine Monophosphate	11.6	306.04	613.1	920.16
Uridine Monophosphate	11.6	323.02	647.06	971.1
Inosine Monophosphate	11.7	347.03	695.09	
5-Methylthioadenosine	11.9	296.08	593.18	
3-Phosphoglyceric acid	13.6	184.9	370.9	556.9
Phosphoenolpyruvate	14.4	166.9	334.9	502.9
Adenosine Diphosphate	14.5	426.02	853.06	
Fructose Bisphosphate	14.7	338.9	678.9	
Ribulose 1,5 Bisphosphate	14.9	308.9	618.9	
Deoxy-Adenosine triphosphate	15.2	489.9	980.9	

^a^ A 5 μL sample for each of these standard metabolites with approx. 0.1 mM were injected into a reverse phase HPLC column.

^b^ The retention time obtained are based on the reverse phase column and ion-paring agents used in the HPLC method as described in the methods section.

In a typical metabolite identification workflow, the presence of multimer adducts are considered to avoid erroneous identification of putative metabolites. Although the identification and quantification of multimers are routine in proteomics based on the exact mass, retention time and characteristics of the monomer ion peak, this is not the case in metabolomics. We believe that since the information of these adducts are not described effectively in databases, most of the software tools for identification can miss to annotate them. This opens up a requirement for the addition of new paradigms into the annotation and quantitation software tools to utilize the information obtained through the evaluation of these multimers. Our study and the data on these standard metabolites can assist in the process.

### Presence of multimer adducts in samples

In order to characterize the multimer adducts, we used time course isotopic label incorporation measurements of ^13^C labeled substrate in three different biological systems. We performed experiments on the cyanobacterial strain PCC 7002. Cyanobacterial systems are photosynthetic workhorse used to produce various high value chemicals and biofuels [32,33]. The other two datasets obtained from repositories used the CD34+ and MGA3 systems [13,24]. The human-stem-cell derived erythrocytes (CD34+) are not only an excellent model system to study the cell physiology of erythrocytes, but also hold promise in therapeutic applications. *B*. *methanolicus* MGA3 is a gram-positive methylotroph which uses methanol as a carbon source to produce high-value chemicals. It can grow at elevated temperature and therefore is an interesting host organism as a microbial cell factory. As described in the methods section the sample preparation and analysis of these three datasets used were very different from each other, which corroborates a wider purview of this study.

For the in-house data using cyanobacterial strains, out of the 24 metabolites listed in Table 1 we observed dimer ions for 7 known (Table 2) and 8 putative (Table 3) compounds. The dimer to monomer ratio varied between the metabolites and the sample type (Table 2). In most of the multimer adducts identified, the dimer to monomer ratio was < 1. Although, in all our sample injections, the multimer peaks were less intense than the monomer peaks, the possibility of a more intense multimer peak will be dependent on experimental conditions (sample concentration and LCMS parameter settings). Using a modified extraction method [14], which ensures efficient extraction of phosphate containing compounds the dimer to monomer ratio for the metabolites PEP and 3PGA in strain PCC 11801 was > 1 (S25 Fig). Therefore, the ratio could be potentially affected by several factors such as the quality of the metabolite extraction, the HPLC method used, and the MS parameters selected. The number of multimer ions detected and the ratio (dimer-monomer) was lower in the strains MGA3 and CD34+ as compared to the data collected in-house for the cyanobacterial samples (Table 2). Similar to the observation in known compounds, in case of all the 8 putative m/z values, the monomer and the dimer peaks overlap closely with respect to the retention time (S26 Fig). In the MS spectrum, the monomer fragment is one of the abundant ions of the dimer spectrum as observed in all the standard injections.

**Table 2:** Dimer to monomer ratio identified in studied datasets. The dimer to monomer ratio was calculated from the intensity/area of dimer and monomer ions identified in each of the studied dataset using XCMS online. The data is presented for the strains, *Synechococcus* sp. PCC 7002 (PCC 7002), *Synechococcus elongatus* PCC 11801 (PCC 11801), *B*. *methanolicus* MGA3 and human stem cell derived erythrocytes (CD34+).

Metabolite	m/z	PCC 7002	PCC 11801	MGA3	CD34+
Phosphoenolpyruvate	166.9	0.56	1.2	ND^a^	ND*^b^
3-Phosphoglyceric acid	184.9	0.25	1.92	0.11	0.22
Hexose 6 Phosphate	259.01	ND	0.41	0.1	0.085
Ribulose 1,5 Bisphosphate	308.9	0.06	0.15	ND^a^	ND* ^b^
Uridine Monophosphate	323.02	0.02	0.42	0.02	ND* ^b^
Sucrose	341.1	0.32	0.46	ND^a^	ND* ^b^
Adenosine diphosphate	426.02	0.04	0.17	0.04	ND* ^b^

^a^ND represents the metabolites which were not identified

^b^ND* represents metabolites which are not detected when negative polarity is specified in the script.

**Table 3:** List of putative compounds with their multimer adducts obtained in the in-house dataset. The list of putative m/z values for the monomer and dimer peaks identified in an in-house sample from strain *Synechococcus elongatus* PCC 11801. The monomer and dimer m/z identified were not annotated.

No.	RT (min)	m/z	
		[M-H]^-^	[2M-H]^-^
1	9.8	245.10	491.21
2	10.5	277.08	555.17
3	15.3	386.23	771.47
4	16.2	254.12	509.24
5	16.8	293.17	587.35
6	18.3	353.19	635.47
7	18.8	287.24	573.48
8	19.1	255.23	511.47

Our key observation here is that these multimer ion adducts are ubiquitous in metabolomics datasets irrespective of how the data was collected (described earlier about the variation in datasets used). All the multimer adducts identified from the public domain data were either not annotated or reported as an unidentified metabolite feature in the original report. Although it did not affect the results of the presented in those studies it unveils an area in the field, which needs to be further characterized.

### Correlation of Mass Isotopomer Distribution between monomers and dimer ion adducts

Metabolic flux analysis has a stringent requirement for accurate MIDs of intermediate/terminal metabolites [34]. However, depending on the quality of the peaks, the density of m/z at a particular retention time, and the number of carbon atoms in the molecule, it can be strenuous to obtain the required MIDs in one run. In such a situation, the alternative is to repeat the sample analysis with a modified HPLC method to decrease the matrix effect and/or to choose specifically targeted analysis such as MRM/PRM. This demand for a larger investment of time and therefore the possibility to obtain the required MIDs of a metabolite without repeating the analysis will always be preferred. Here, we present MIDs of many dimer adducts identified in various samples (cyanobacteria, methylotrophs, and stem cell-derived erythrocytes) using different chromatographic separation methods and mass detectors of different makes. The MID obtained for these adducts are then used to calculate the MIDs of the monomer ions which can be used for flux estimations in ^13^C-MFA.

**Fig 1:**
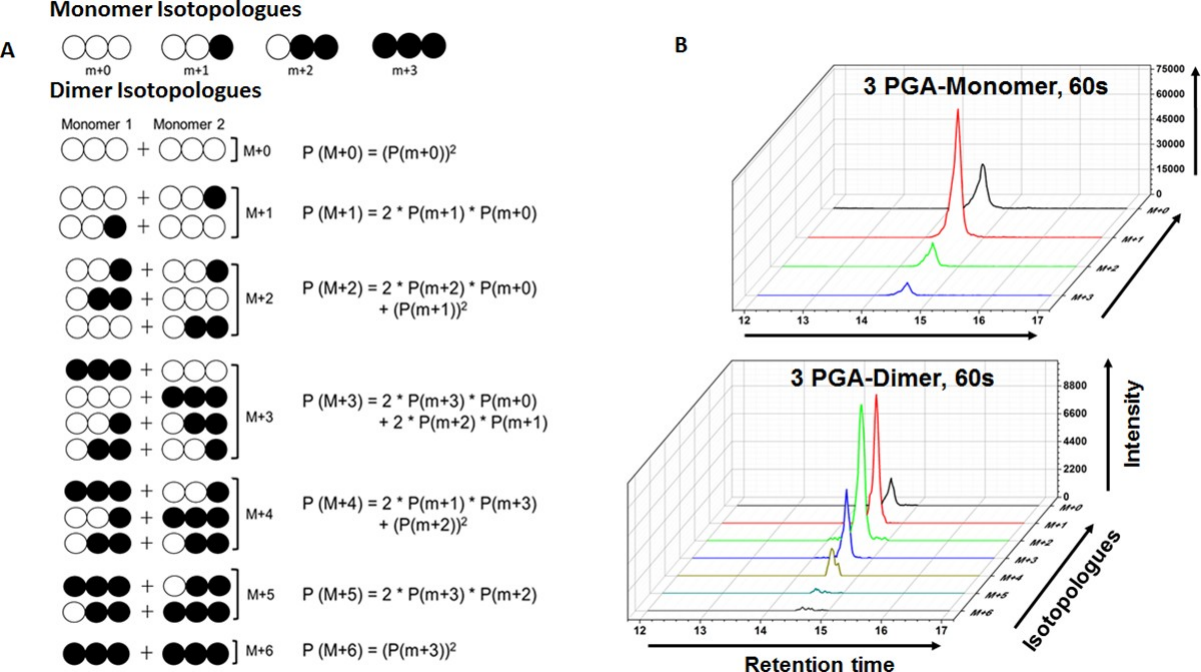
Mass Isotopologue Distribution of the monomer and dimer units of a 3 carbon molecule. A) Schematic representation of the monomer isotopologues and the probable combinations of monomer units to form the dimer isotopologues and the mathematical relationship between the monomer and dimer isotopologus. The labeled carbon with ^13^C is shown using filled circles B) Extracted ion chromatogram (XIC) of the isotopologues in the monomer ions (upper panel) and dimer ions (lower panel) of 3 phosphoglyceric acid (3 PGA) observed in a sample from strain *Synechococcus* sp. PCC 7002 at 60s after the labeled ^13^C sodium bicarbonate was added to the culture.

In each of the sample data tested, MIDs of dimers could be quantitated for several metabolites. The distribution in the dimer ions can be attributed to the various possible combinations from the monomer units. For example in Fig 1A one such distribution of monomer and dimer isotopologues for a 3 carbon compound is illustrated. The distribution for the dimer units (M+0, M+1, M+2, M+3, M+4, M+5, and M+6) can be calculated using the MIDs of the monomer isotopologues considering all the probability of monomer association to form the dimer. In Fig 1B, the monomer and dimer MIDs of 3 phosphoglyceric acid (3 PGA), identified in a 60s sample (sample collected 60s after the addition of ^13^C labeled bicarbonate to an exponentially growing culture of cyanobacteria PCC 7002) is shown. All the isotopologues for both monomer and dimer ions were identified for the metabolite 3PGA and therefore MIDs of both the ions could be measured. Using the formula shown in Fig 1A, the monomer MIDs can be calculated from the measured MIDs of the dimer. This requires solving an over-determined system using the least square regression analysis in MATLAB.

**Fig 2:**
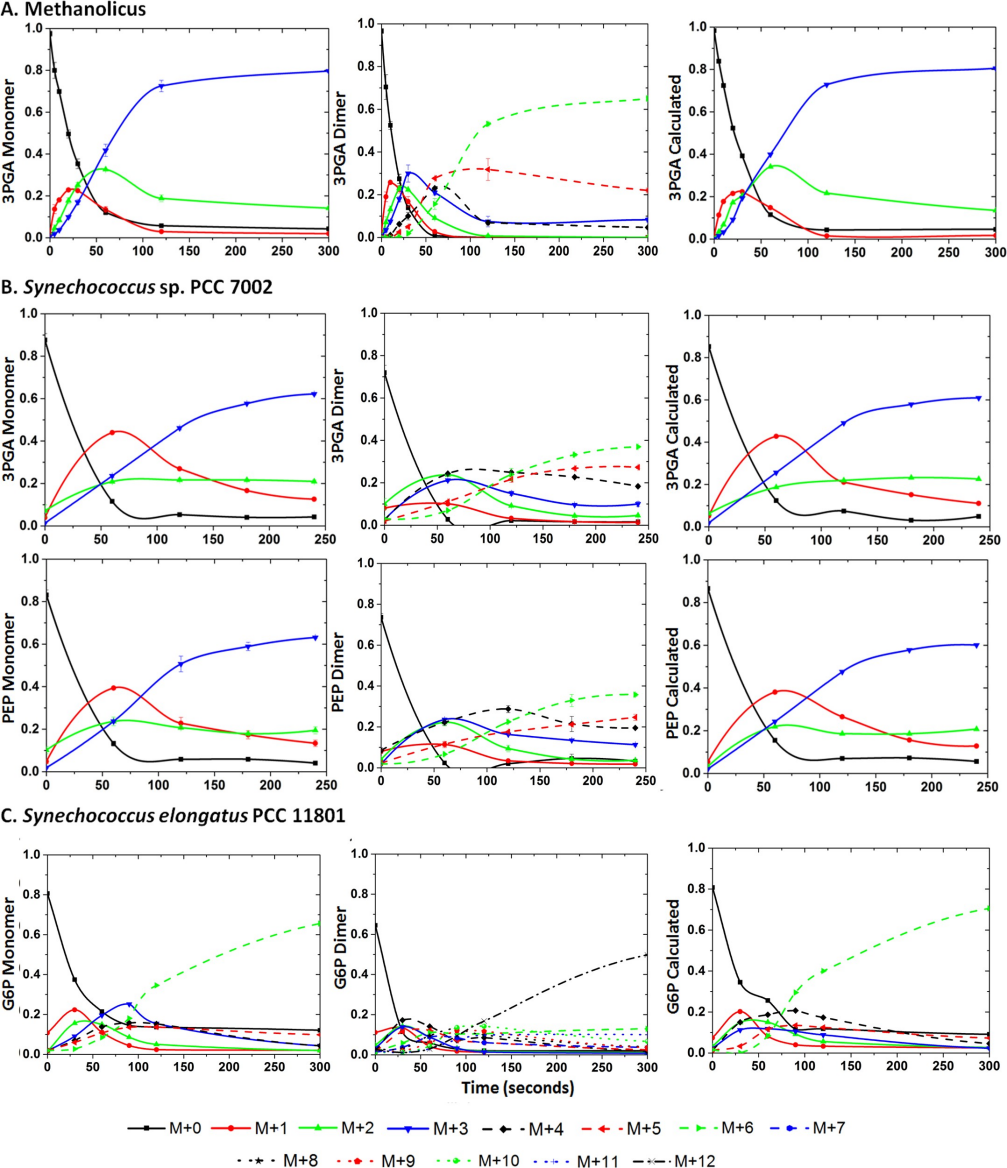
Mass isotopologue distribution of ion adducts quantitated from a time course measurement of ^13^C labeling in various organisms. The MID of monomer ions (panels in the left column), dimer ions (panels in the middle column), and the calculated MIDs of the monomer from the dimer measurements (panels in the right column) are shown for all the metabolites presented. A) Measured MID of monomer and dimer ion adducts of 3 phosphoglyceric acid (3 PGA) from the strain *B*. *methanolicus* MGA3. B) Measured MID of monomer and dimer ion adducts of 3 phosphoglyceric acid (3 PGA) and phosphoenolpyruvate (PEP) from the strain *Synechococcus* sp. PCC 7002. C) Measured MID of monomer and dimer ion adducts of glucose 6 phosphate (G6P) from the strain *Synechococcus elongatus* PCC 11801.

**Fig 3:**
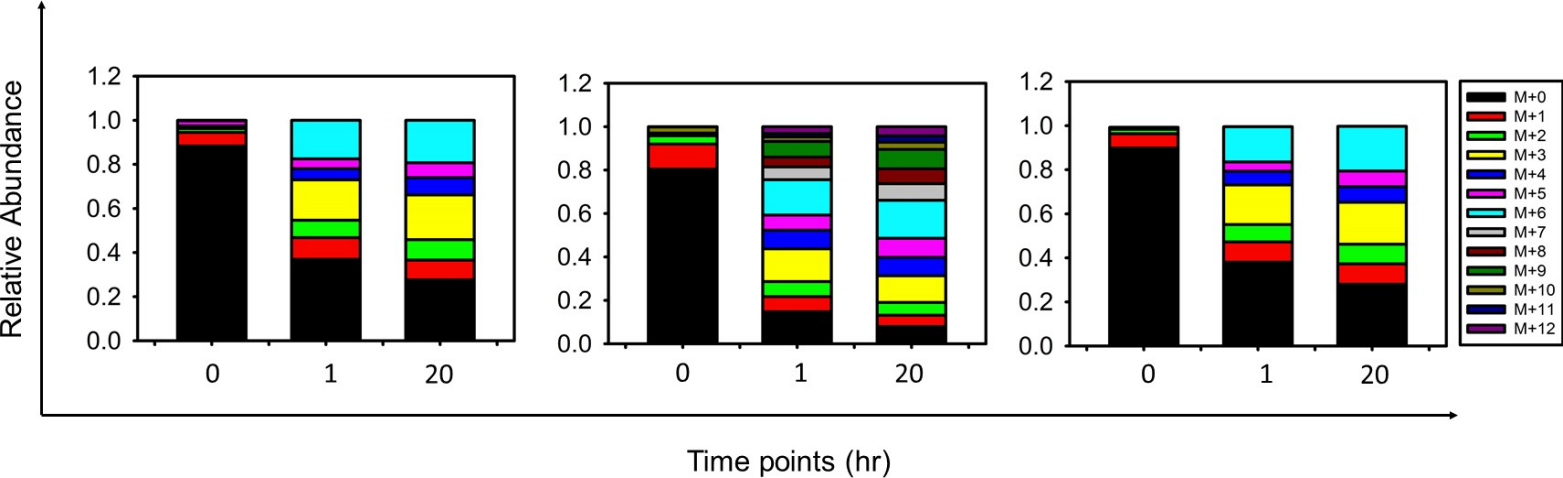
Mass isotopologue distribution of ion adducts quantitated from a published study on Red Blood Cells (Reticulocytes) with ^13^C labeling monitored at 1 hour and 20 hours after introduction of the tracer. The MIDs of monomer ions (panel on the left), dimer ions (middle panel), and the calculated MIDs of the monomer from the dimer measurements (panel on the right) are shown for the metabolite fructose 6 phosphate (F6P). The monomer distribution was presented in the published study and the dimer distribution was quantitated by us using the data provided.

**Fig 4:**
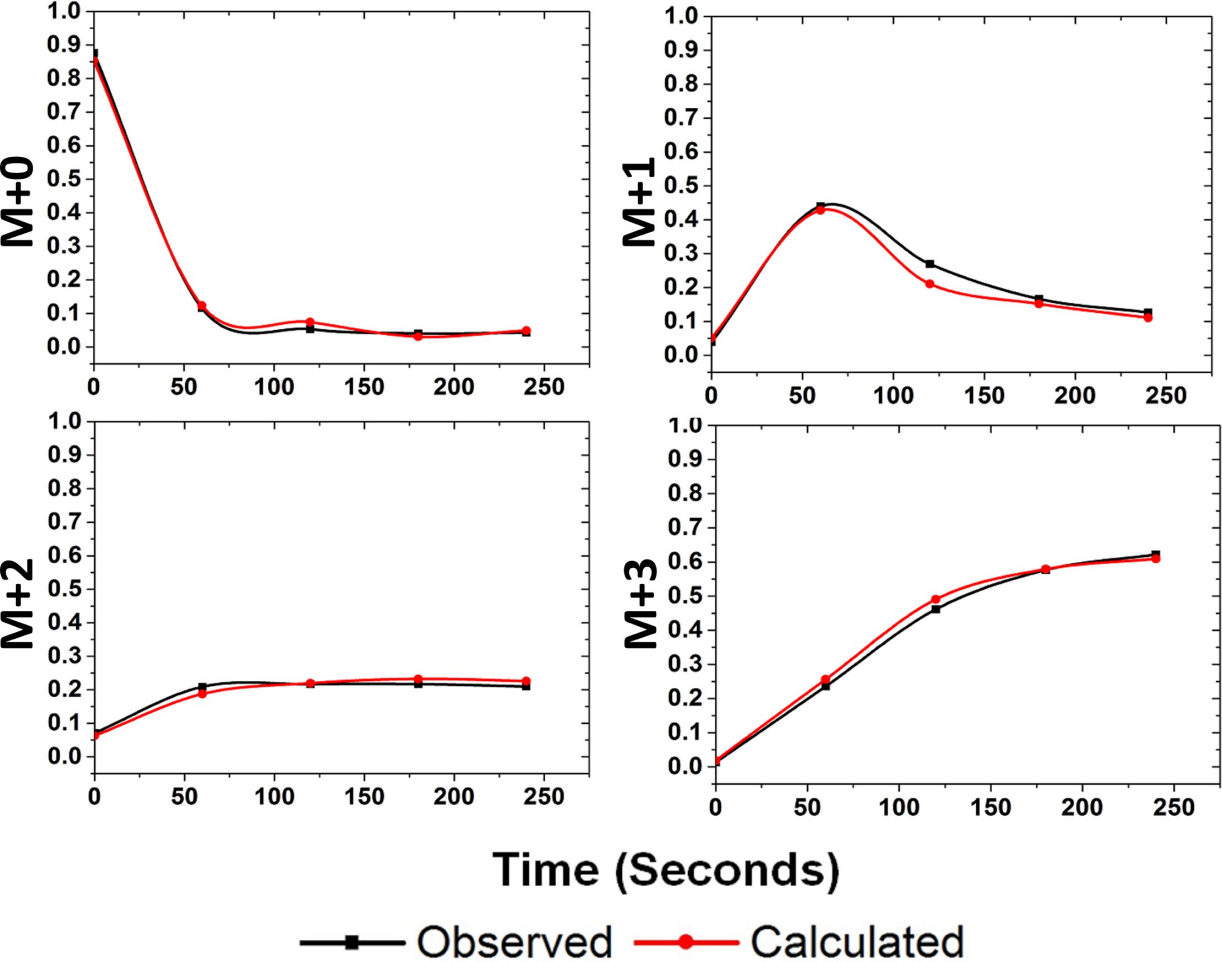
Overlay plot for the mass isotopologues of monomer ion of 3PGA quantitated and calculated for dataset from *Synechococccus* sp. PCC 7002. The mass isotopologues for 3PGA, M0, M1, M2 and M3 quantitated from the monomer peak (black trace) and calculated (red trace) from the MIDs of the corresponding dimer ions. The monomer and the dimer MID pattern for the metabolite are shown in Fig 2.

**Fig 5:**
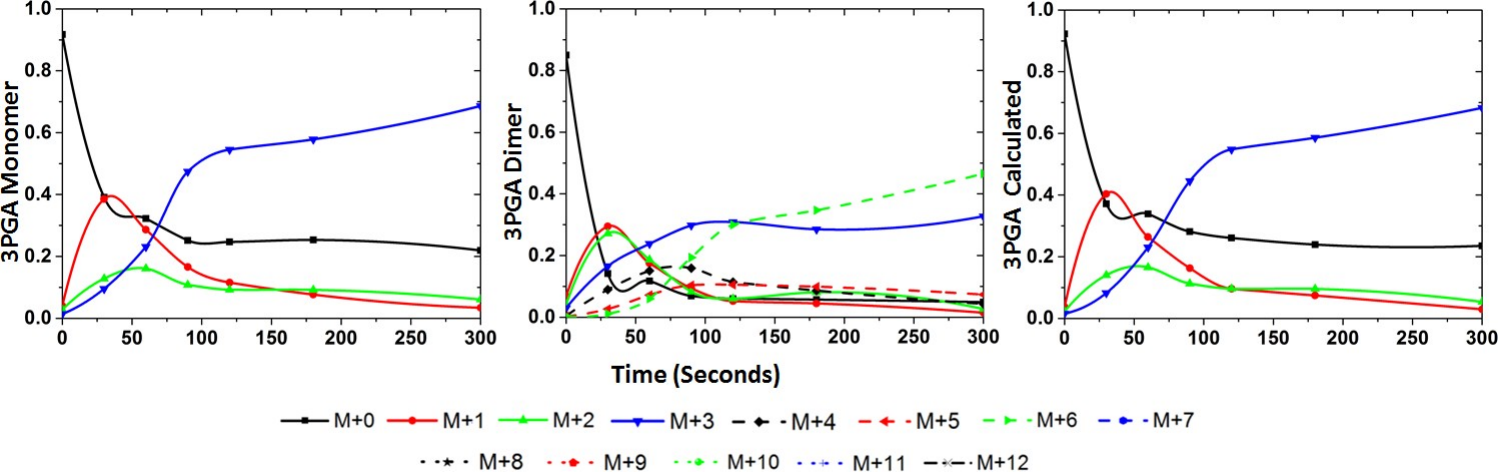
Mass isotopologue distribution ion adducts of 3PGA, quantitated from a time course measurement and the MIDs of dimer ions calculated from monomer ions. The MID of monomer ions (panels in the left column), dimer ions (panels in the middle column), and the calculated MIDs of the monomer from the dimer measurements (panels in the right column) are shown for 3 phosphoglyceric acid (3 PGA) from the strain *Synechococccus elongatus* PCC 11801. In this case the peak intensity observed for the dimer ions were higher than that for the monomer ions.

The MIDs of monomer, dimer, and monomer calculated from dimer ions for different metabolites quantitated from the various datasets studied are shown in Figs 2 and Fig 3. The data from CD34+ cells consisted of two time points after the addition of ^13^C substrate and is therefore presented as a stacked bar format in Fig 3. In both the figures, the calculated monomer MIDs correlated well with the measured values for metabolites. Overlay plot of the quantitated isotopologues and those calculated from the dimer ions are shown in Fig 4. The chi square analysis on the calculated and measured values were non-significant. These measurements have been demonstrated for compounds of different carbon length (e.g., G6P/F6P, 3PGA and PEP). The correlation of the measured and calculated distributions were good for dimers calculated from monomer ions as well (S26 Fig and S27 Fig). As described earlier in Table 2 we did observe cases where the dimer to monomer ratio was >1, for a few metabolites. In Fig 5, we display one such example from the strain PCC 11801 for the metabolite 3PGA where the dimer to monomer ratio at 0s time point is 1.92. Based on Figs 2–5, it is clear that while the relative abundance of dimer to monomer may vary depending on the type of sample and the experimental conditions, the accuracy of MIDs of monomer ions calculated from dimers always remained high. This analysis suggests the utilization of multimer adducts in obtaining the MIDs of the monomer ions. In case of the MFA, where the quality of the MIDs and the replicate measurements used ensures the accuracy of measured fluxes this application will be particularly handy.

## Conclusion

A novel application of using the MIDs of dimer ion adducts of metabolites from LC/MS data to obtain the MIDs of monomer units has been developed. This method involves the identification and quantification of MIDs of the dimer adduct followed by calculation/deconvolution of the monomer MIDs by solving an overdetermined system using least square regression analysis in MATLAB. This methodology was applied to diverse biological systems such as cyanobacteria, methylotrophs, and human stem cell-derived erythrocytes. The datasets used were collected using various LC and MS methods which showcases the wider application of this study. This approach has been proven useful in calculating reliable MIDs of the monomer units from the dimer measurements and is anticipated to be useful for any kind of isotope labeling studies. The application of this method will be of utmost use when the multimer ions are detected for a metabolite where the peak for the monomer ions cannot be quantitated or has conflicts for one or more of the monomer isotopologues. When both the monomer and dimer data are of comparable quality, the latter may be used as an additional technical replicate. We also provide a data for 24 metabolite standards for which the multimer (dimer and trimer) peaks are identified. The information will be useful for metabolite identification softwares to avoid mis-annotations.

## Supporting information

S1 Table List of parameters from various software programs optimized for individual dataset. (DOCX)

S1 Text Matlab code file for the calculation of mass isotopologue distribution (MIDs) of monomers from MIDs of dimers. (TXT)

S1 Fig Multimer ion adducts observed in an injection of a pure standard compound N-Acetyl D Glucosamine. (PDF)

S2 Fig Multimer ion adducts observed in an injection of a pure standard compound sucrose. (PDF)

S3 Fig Multimer ion adducts observed in an injection of a pure standard compound uridine. (PDF)

S4 Fig Multimer ion adducts observed in an injection of a pure standard compound Glucono 1, 5 Lactone. (PDF)

S5 Fig Multimer ion adducts observed in an injection of a pure standard compound gluconic acid. (PDF)

S6 Fig Multimer ion adducts observed in an injection of a pure standard compound shikimate. (PDF)

S7 Fig Multimer ion adducts observed in an injection of a pure standard compound inosine. (PDF)

S8 Fig Multimer ion adducts observed in an injection of a pure standard compound quinate. (PDF)

S9 Fig Multimer ion adducts observed in an injection of a pure standard compound dihydroorate. (PDF)

S10 Fig Multimer ion adducts observed in an injection of a pure standard compound glucose 6 phosphate. (PDF)

S11 Fig Multimer ion adducts observed in an injection of a pure standard compound tryptophan. (PDF)

S12 Fig Multimer ion adducts observed in an injection of a pure standard compound tryptophan. (PDF)

S13 Fig Multimer ion adducts observed in an injection of a pure standard compound sedoheptulose 7 phosphate. (PDF)

S14 Fig Multimer ion adducts observed in an injection of a pure standard compound ribulose 5 phosphate. (PDF)

S15 Fig Multimer ion adducts observed in an injection of a pure standard compound deoxy-cytidine monophosphate. (PDF)

S16 Fig Multimer ion adducts observed in an injection of a pure standard compound uridine monophosphate. (PDF)

S17 Fig Multimer ion adducts observed in an injection of a pure standard compound inosine monophosphate. (PDF)

S18 Fig Multimer ion adducts observed in an injection of a pure standard compound 5-methylthioadenosine. (PDF)

S19 Fig Multimer ion adducts observed in an injection of a pure standard compound 3-phosphoglyceric acid. (PDF)

S20 Fig Multimer ion adducts observed in an injection of a pure standard compound phosphoenolpyruvate. (PDF)

S21 Fig Multimer ion adducts observed in an injection of a pure standard compound adenosine diphosphate. (PDF)

S22 Fig Multimer ion adducts observed in an injection of a pure standard compound fructose 1,6 bisphosphate. (PDF)

S23 Fig Multimer ion adducts observed in an injection of a pure standard compound ribulose 1,5 bisphosphate. (PDF)

S24 Fig Multimer ion adducts observed in an injection of a pure standard compound deoxy adenosine triphosphate. (PDF)

S25 Fig Overlay plots of extracted ion chromatograms from putative monomer-dimer pairs observed in the strain *Synechococcus elongatus* PCC 11801. (PDF)

S26 Fig Mass isotopologue distribution ion adducts of 3PGA, quantitated from a time course measurement and the MIDs of dimer ions calculated from monomer ions. (PDF)

S27 Fig Overlay plot for the mass isotopologues of dimer ion of 3PGA quantitated and calculated for dataset from *Synechococccus elongatus* PCC 11801. (PDF)

S28 Fig Comparison of monomer MIDs (observed) and monomer MIDs (calculated from dimer MIDs) of 3 Phosphoglyceric acid after correcting for natural labeling. The area obtained for the monomer ions were corrected for natural labeling. The area obtained for the dimer ions were used for calculation and the monomer MIDs calculated was corrected for natural labeling.
(PDF)

## Abbreviations

3-PGA: 3 Phosphoglyceric acid
ADP: Adenosine diphosphate
F6P: Fructose 6-phosphate
G6P: Glucose 6-phosphate
MID: Mass Isotopologue Distribution
PEP: Phosphoenolpyruvate
RuBP: Ribulose 1, 5-bisphosphate
UMP: Uridine monophosphate
