# Supplementary material for: Mass Isotopologue Distribution of dimer ion adducts of intracellular metabolites for potential applications in 13C Metabolic Flux Analysis
Source: PLoS One. 2019 Aug 21;14(8):e0220412. doi: 10.1371/journal.pone.0220412 (PMC6703694; doi:10.1371/journal.pone.0220412)
Supplement: S1 Table — (DOCX) [file pone.0220412.s001.docx]

**S1 Table: Parameters of the various software programs used for the datasets studied after a systematic optimization process.**

| **Parameters** | | **PCC 7002^a^** | **Reticulocytes^a^** | **Methanolicus^a^** |
| --- | --- | --- | --- | --- |
| Pre-processing XCMS | Mass error | 25 | 5 | N/A |
|  | mzwid | 0.05 | 0.015 | N/A |
|  | minfrac | 0.1 | 0.1 | N/A |
| geoRge | fc threshold | 1.5 | 1.5 | N/A |
|  | p-value threshold | 0.05 | 0.05 | N/A |
|  | PuInc limit | 500 | 4000 | N/A |
|  | Basepeak mass error | 25 | 15 | N/A |
|  | Basepeak min intensity | 1000 | 2000 | N/A |
| DynaMet | maxMzDifferencePairfinder | N/A | N/A | 0.01 |
|  | mz_diff | N/A | N/A | 0.005 |
|  | rt_diff | N/A | N/A | 100 |
|  | common_noise_threshold_int | N/A | N/A | 1000 |
|  | common_chrom_peak_snr | N/A | N/A | 3 |
|  | common_chrom_fwhm | N/A | N/A | 25 |
|  | mtd_mass_error_ppm | N/A | N/A | 15 |
|  | isolation width | N/A | N/A | 0.003 |
|  | max_nrmse | N/A | N/A | 0.8 |
|  | maxMzDifferencePairfinder | N/A | N/A | 0.01 |

^a^ The optimized parameters shown here are only for those programs which were used to analyze a particular dataset.
