# Supplementary figures and images for: Mass Isotopologue Distribution of dimer ion adducts of intracellular metabolites for potential applications in 13C Metabolic Flux Analysis
Source: PLoS One. 2019 Aug 21;14(8):e0220412. doi: 10.1371/journal.pone.0220412 (PMC6703694; doi:10.1371/journal.pone.0220412)

# Glucono-1,5 -Lactone

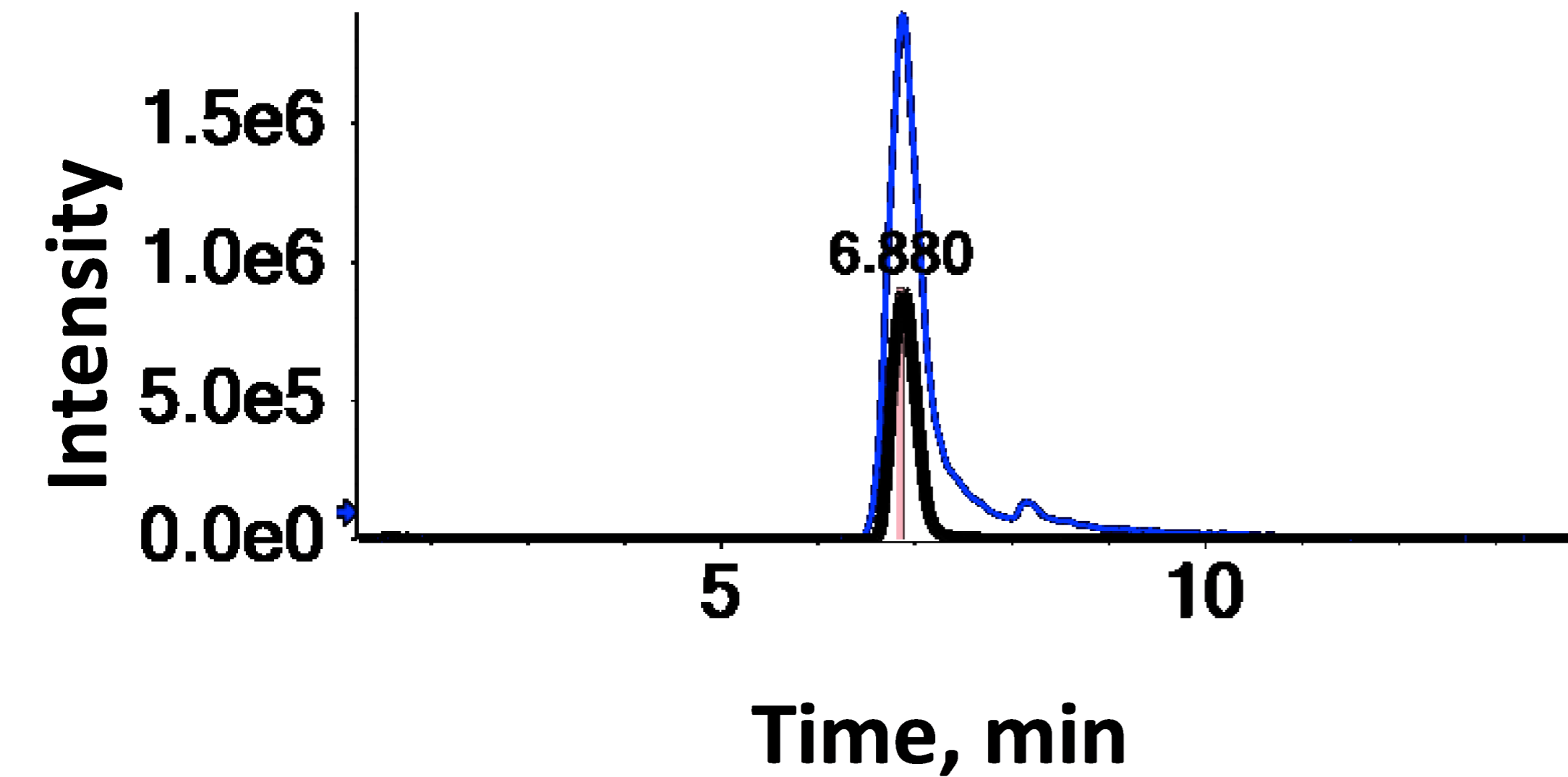

Relative Intensity

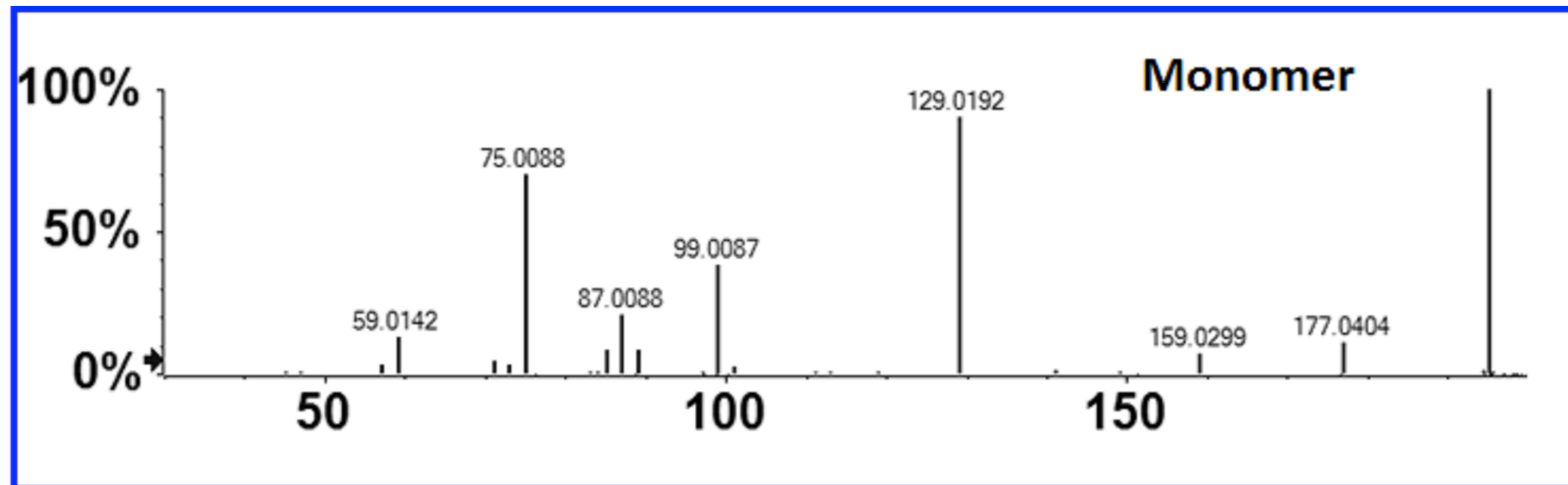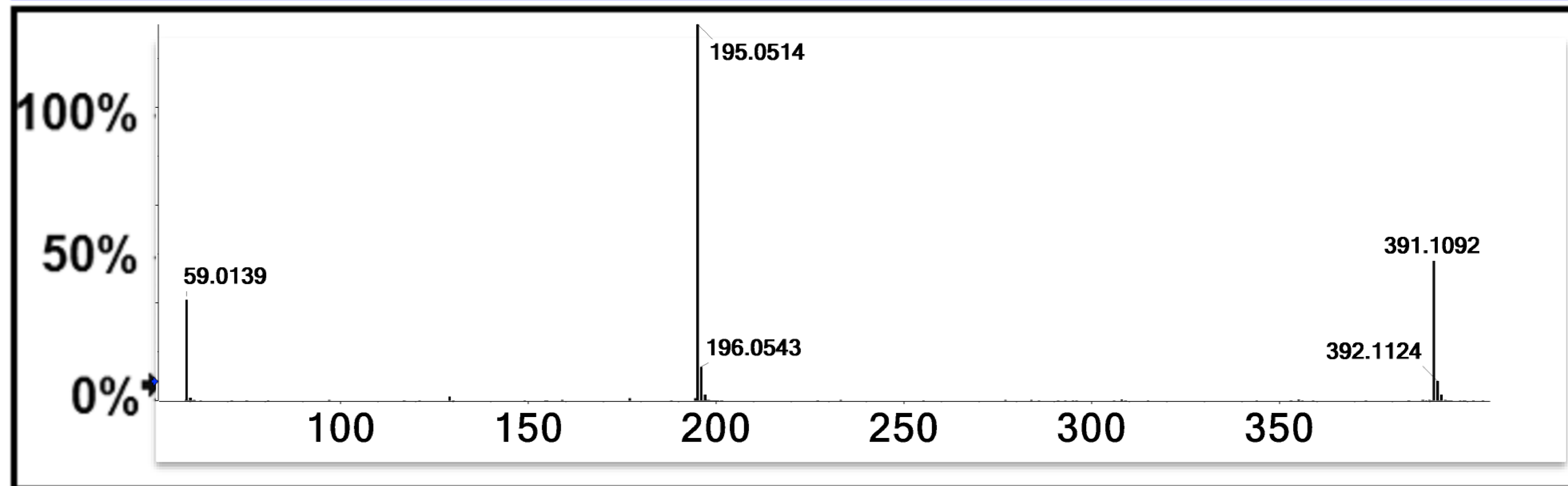

Mass/Charge, Da

Supplement: S4 Fig — (PDF) [file pone.0220412.s006.pdf]

# 5-Methylthioadenosine

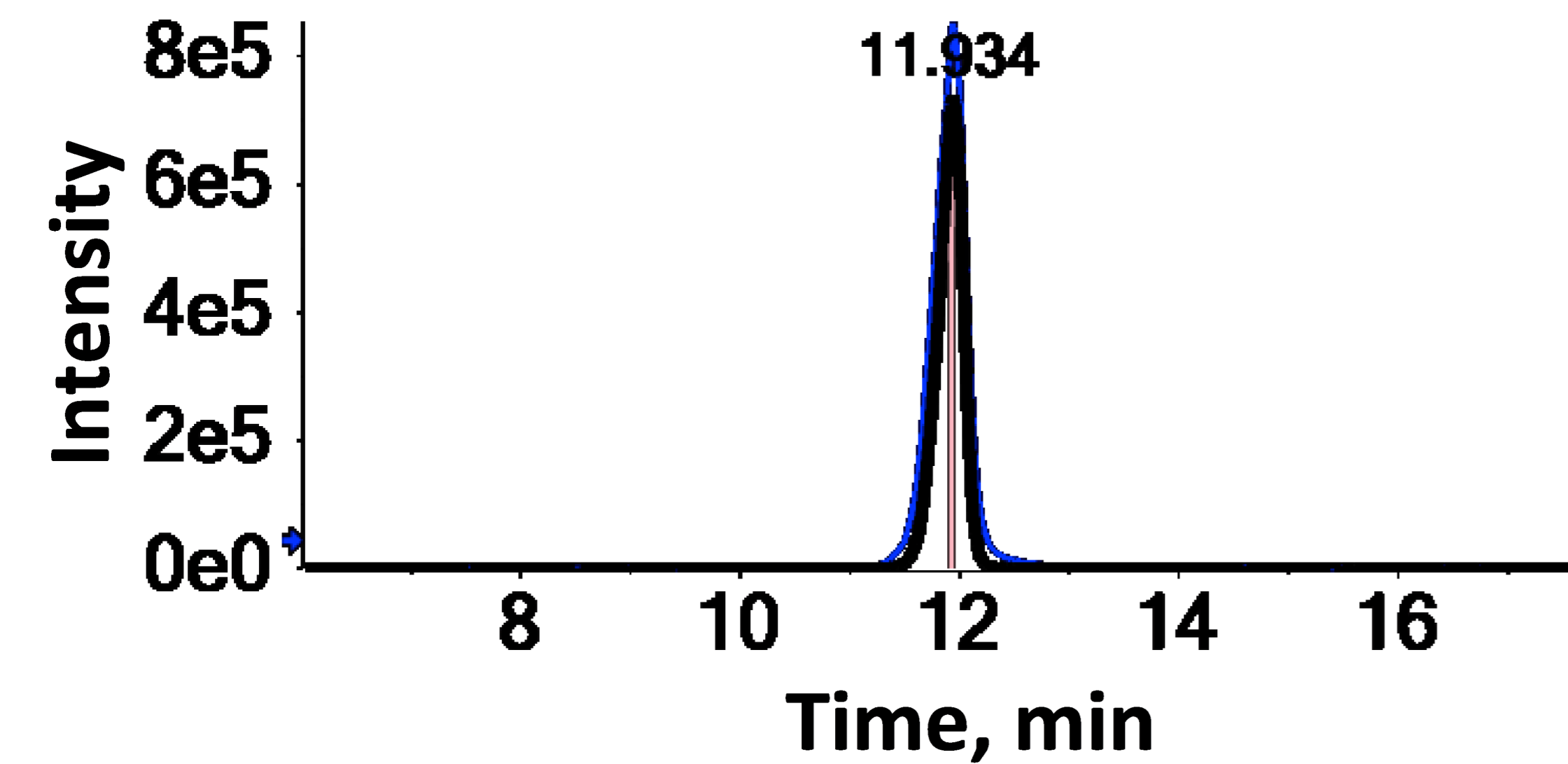

Relative Intensity

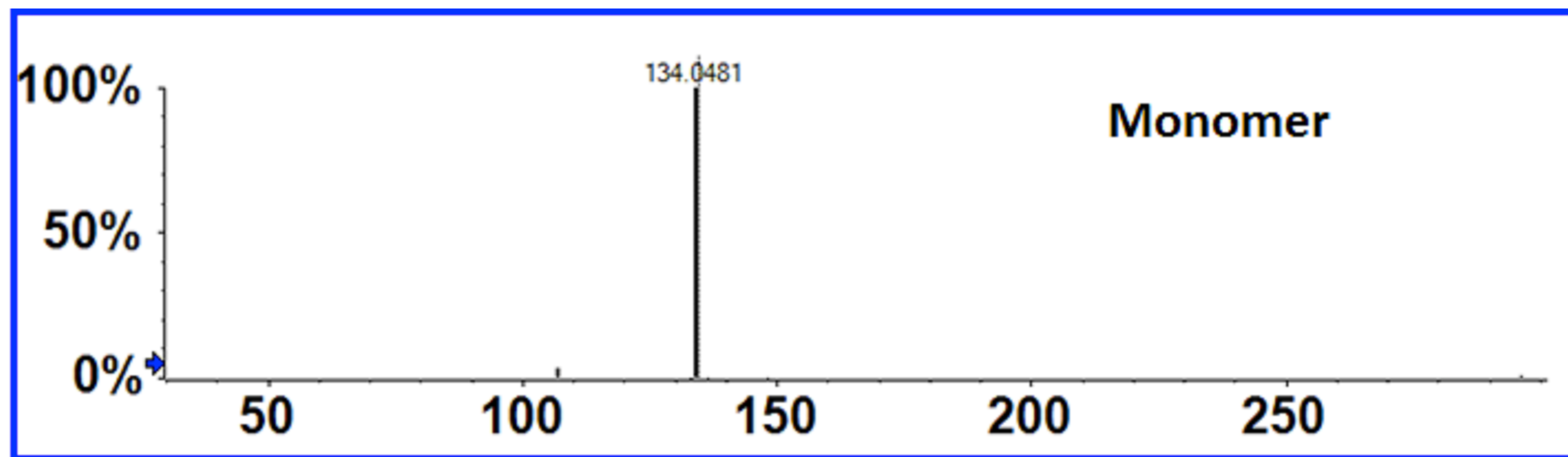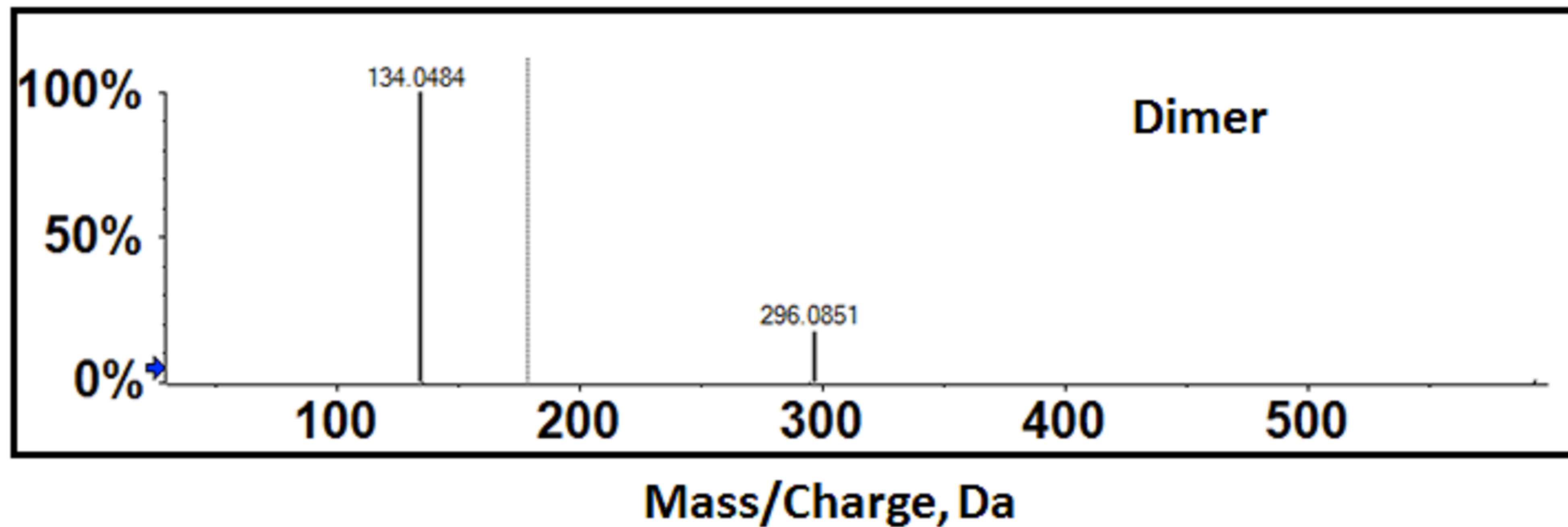

Supplement: S18 Fig — (PDF) [file pone.0220412.s020.pdf]

# Fructose 1,6 biphosphate

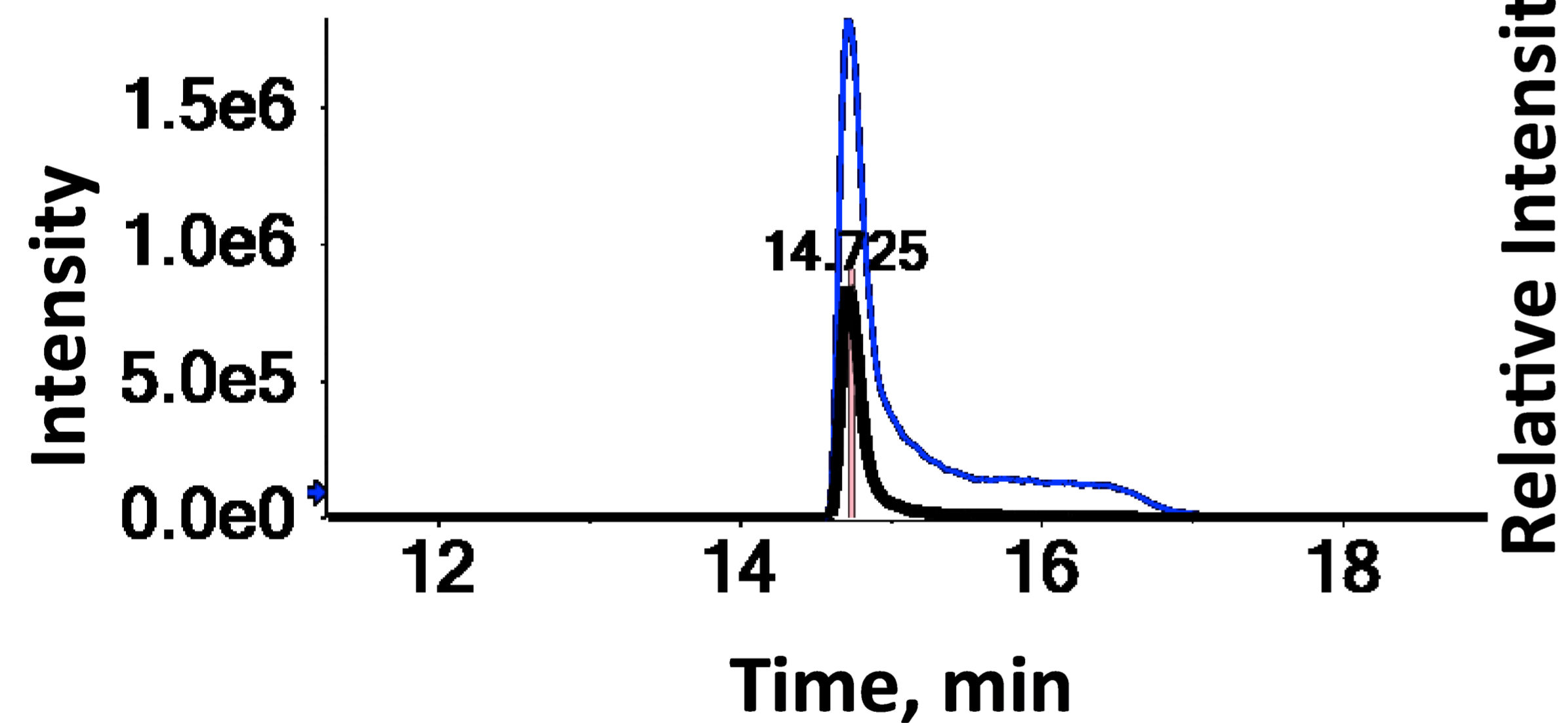

Relative Intensity

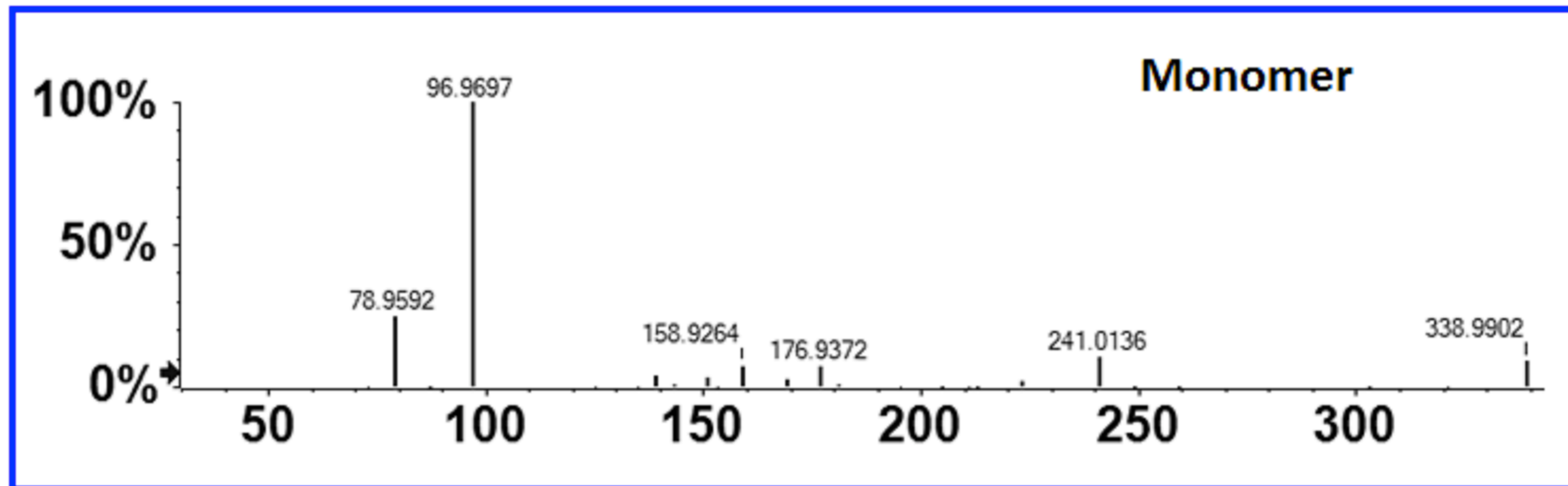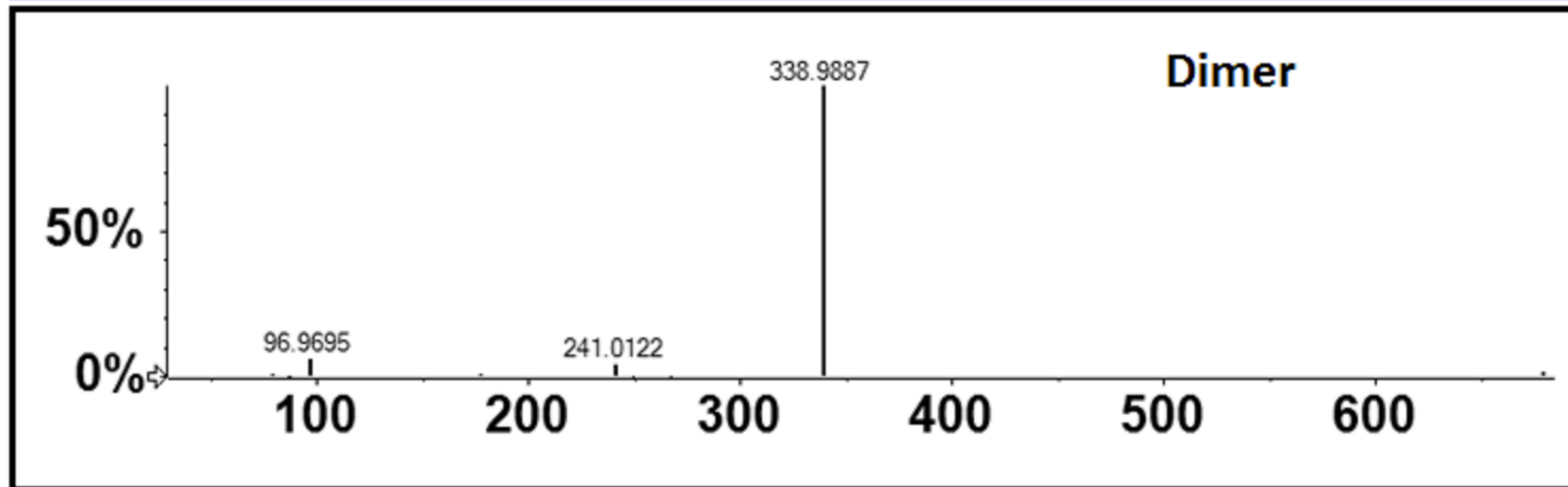

Mass/Charge, Da

Supplement: S22 Fig — (PDF) [file pone.0220412.s024.pdf]

# Deoxy Adenosine triphosphate

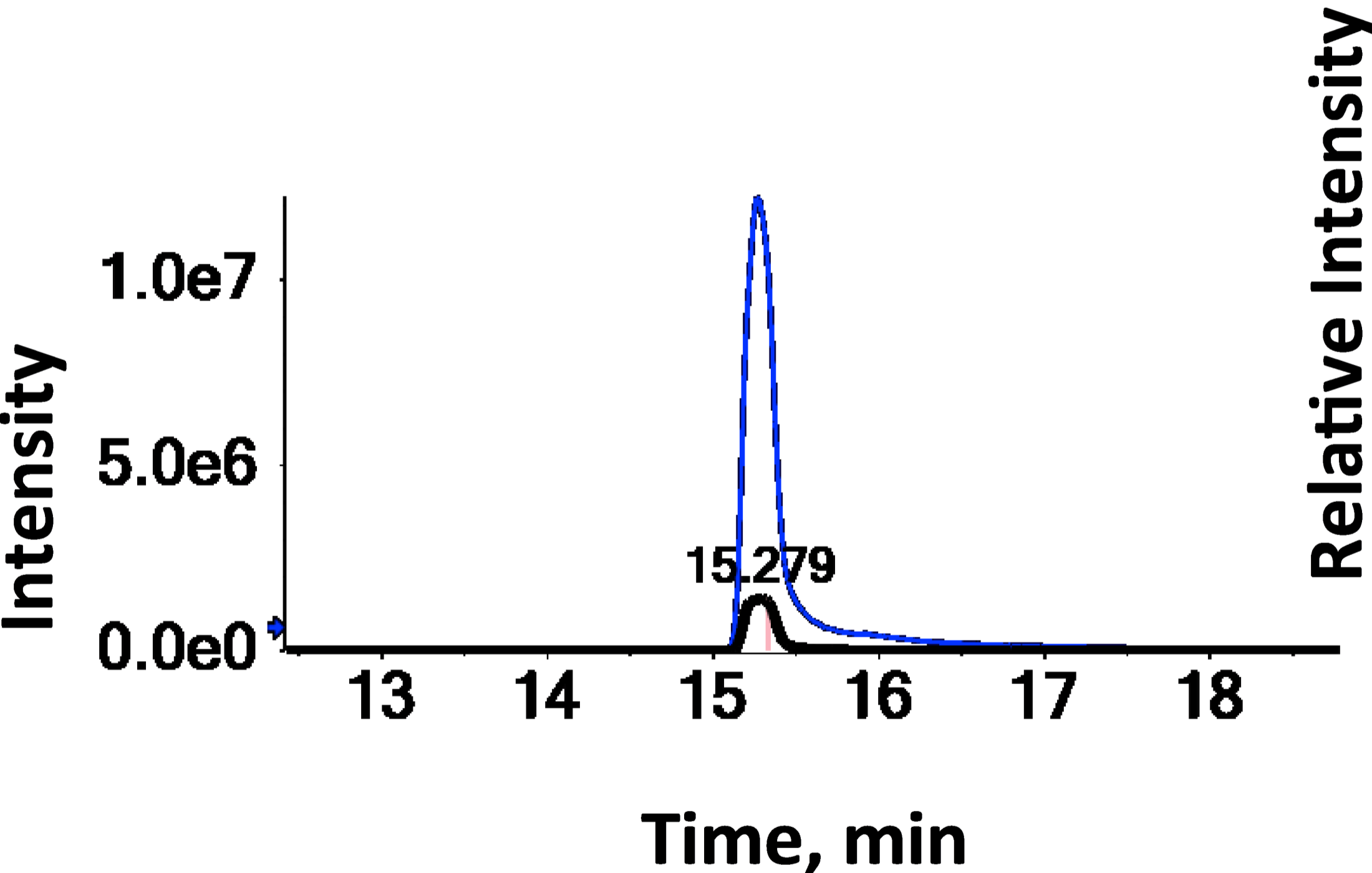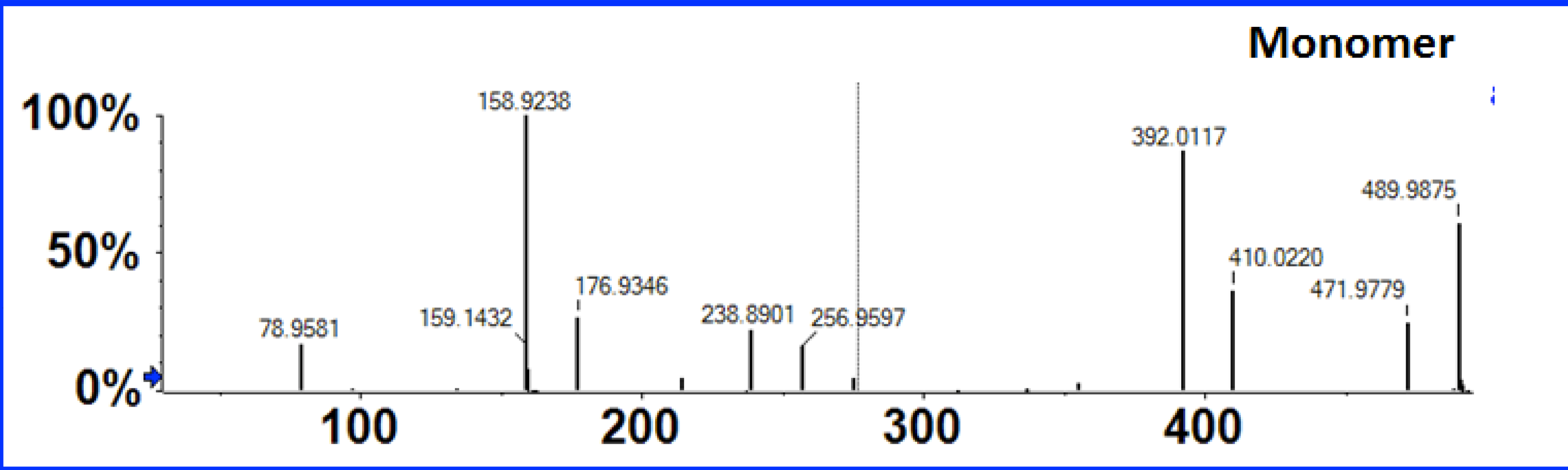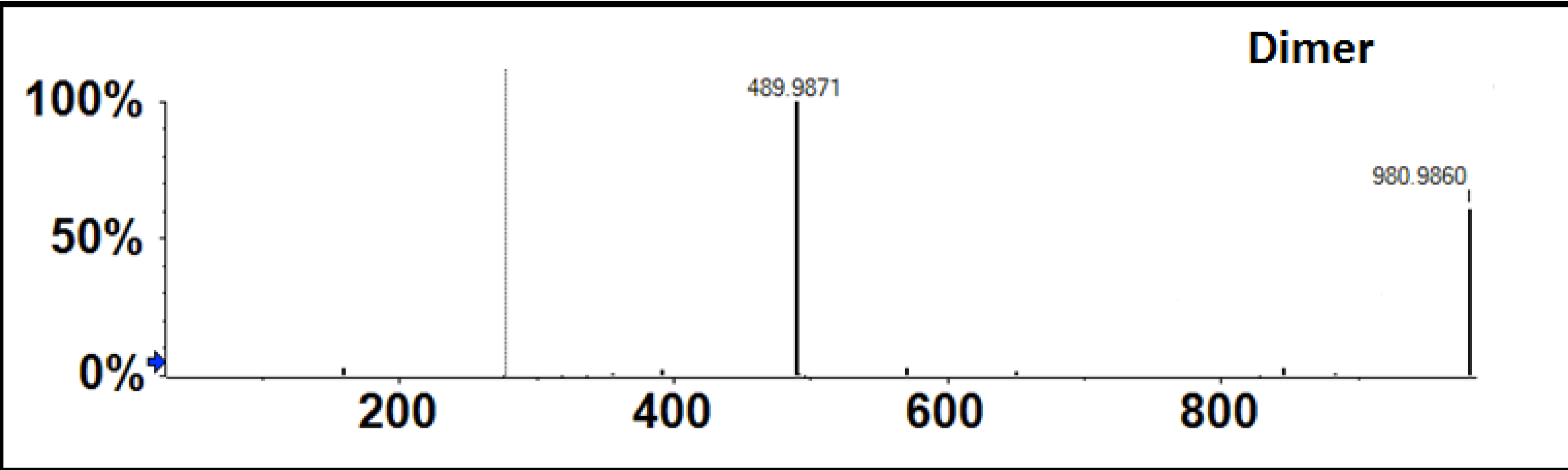

Mass/Charge, Da

Supplement: S24 Fig — (PDF) [file pone.0220412.s026.pdf]

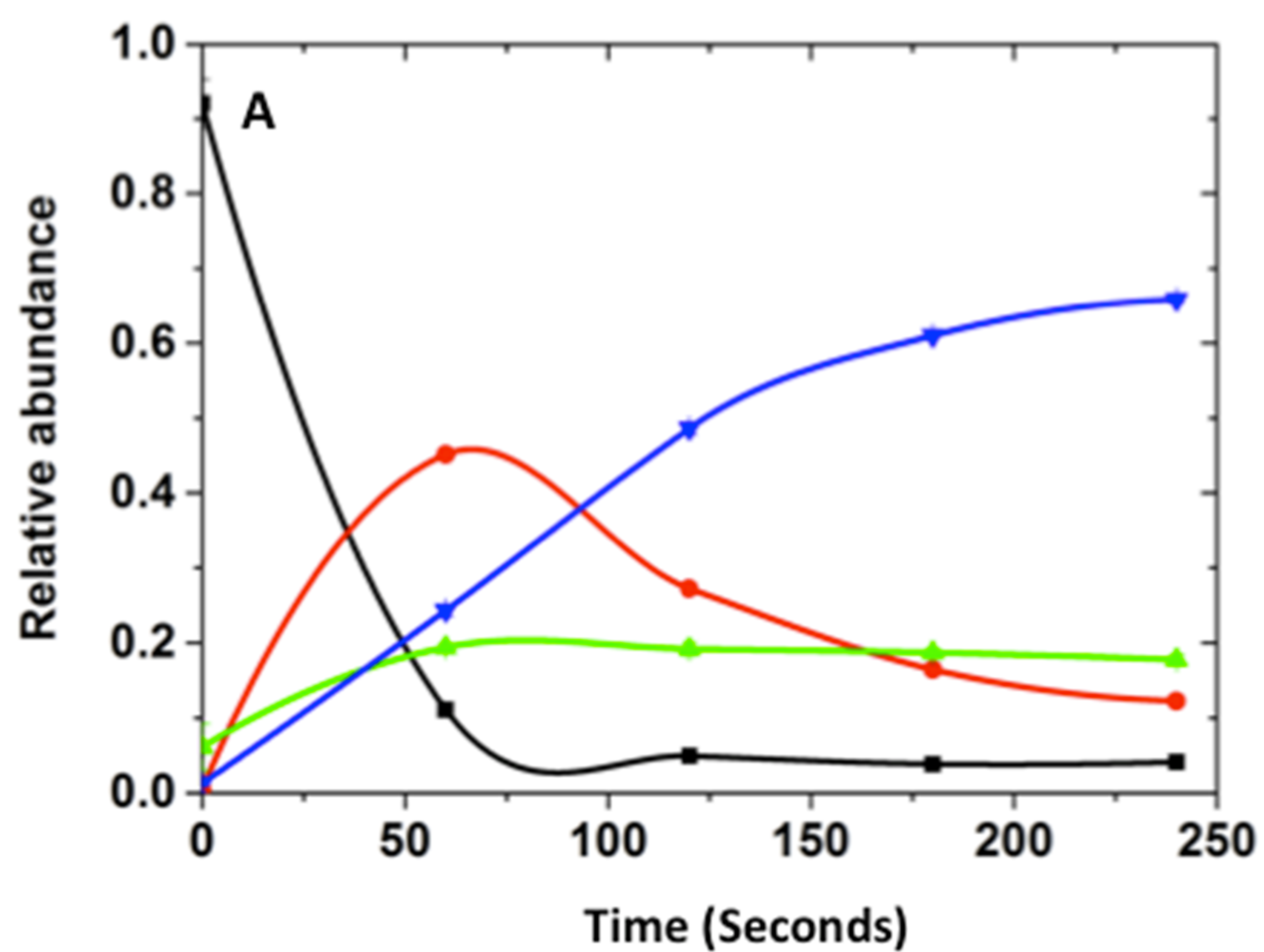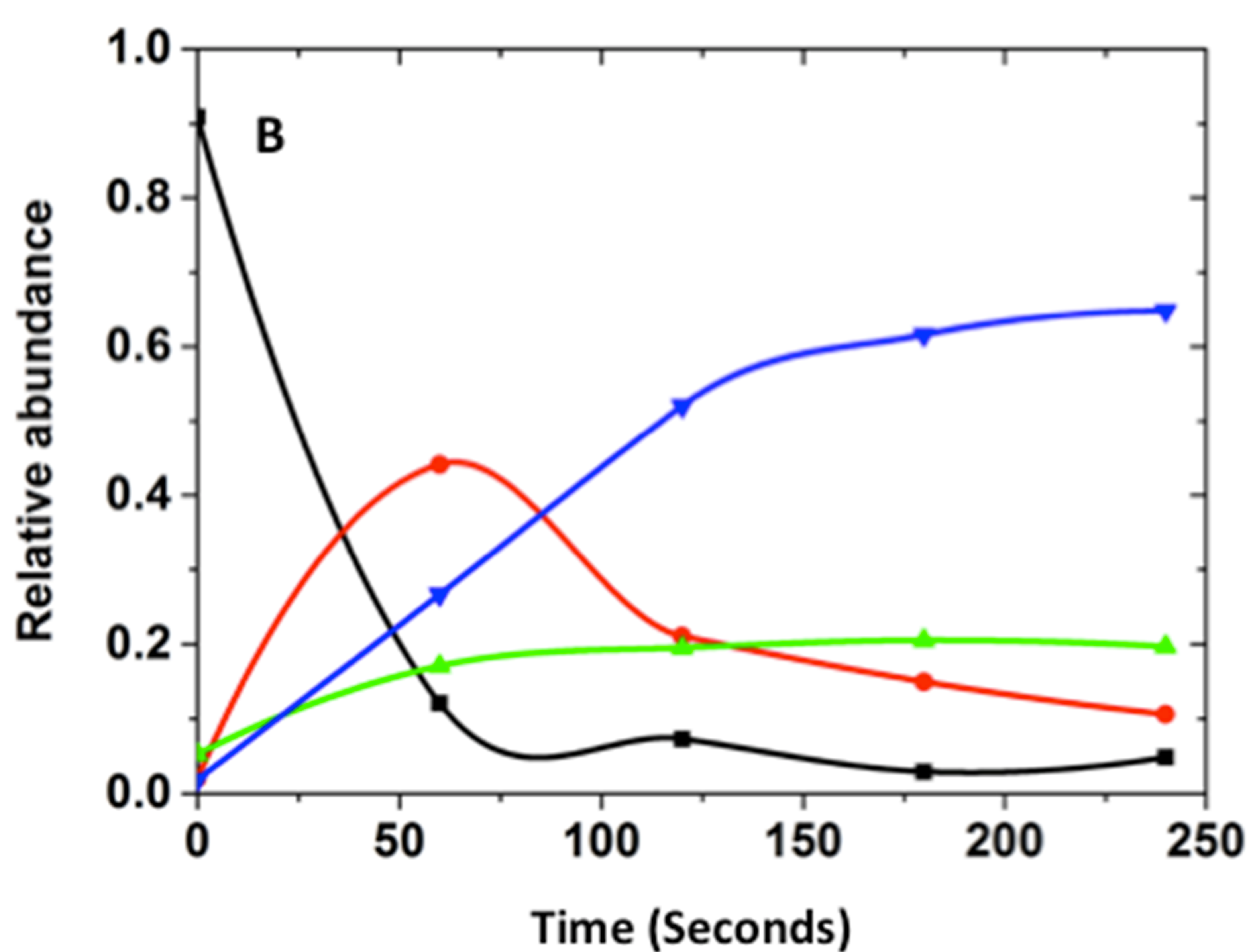

—■— M+0    —●— M+1    —▲— M+2    —▼— M+3

Supplement: S28 Fig — The area obtained for the monomer ions were corrected for natural labeling. The area obtained for the dimer ions were used for calculation and the monomer MIDs calculated was corrected for natural labeling. (PDF) [file pone.0220412.s030.pdf]
