# Supplementary material for: Mass Isotopologue Distribution of dimer ion adducts of intracellular metabolites for potential applications in 13C Metabolic Flux Analysis
Source: PLoS One. 2019 Aug 21;14(8):e0220412. doi: 10.1371/journal.pone.0220412 (PMC6703694; doi:10.1371/journal.pone.0220412)
Supplement: S12 Fig — (PDF) [file pone.0220412.s014.pdf]

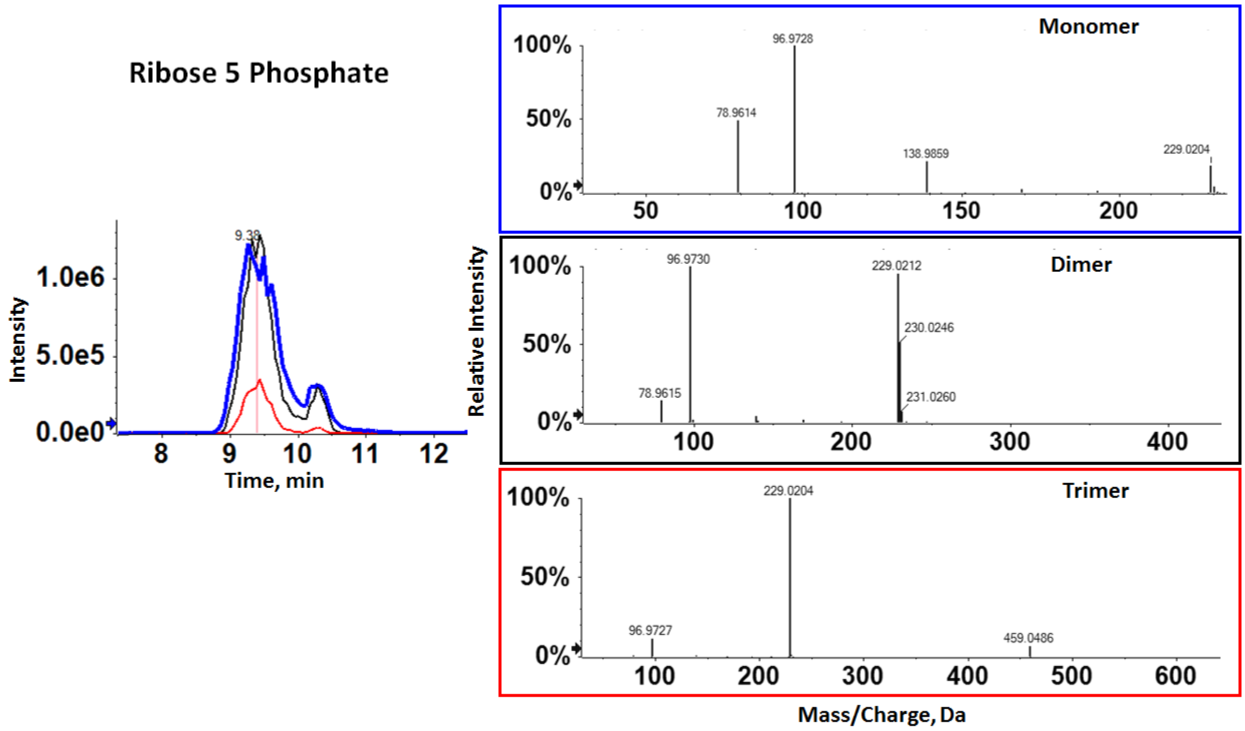

S12 Fig: Multimer ion adducts observed in an injection of a pure standard compound ribose 5 phosphate. The extracted ion chromatograms (XIC) of the monomer and dimer ions is shown in the overlay plot where monomer ions, dimer ions and trimer ions are represented using blue, black and red traces respectively. The MS spectrum of the precursor ion 229.01 (monomer), 459.03 (dimer), and 689.05 (trimer) at a collision energy of -30 eV is presented with relative intensity. We observed the presence of monomer ions in the MS spectrum of the dimer and trimer ions.
