# Supplementary material for: Mass Isotopologue Distribution of dimer ion adducts of intracellular metabolites for potential applications in 13C Metabolic Flux Analysis
Source: PLoS One. 2019 Aug 21;14(8):e0220412. doi: 10.1371/journal.pone.0220412 (PMC6703694; doi:10.1371/journal.pone.0220412)
Supplement: S19 Fig — (PDF) [file pone.0220412.s021.pdf]

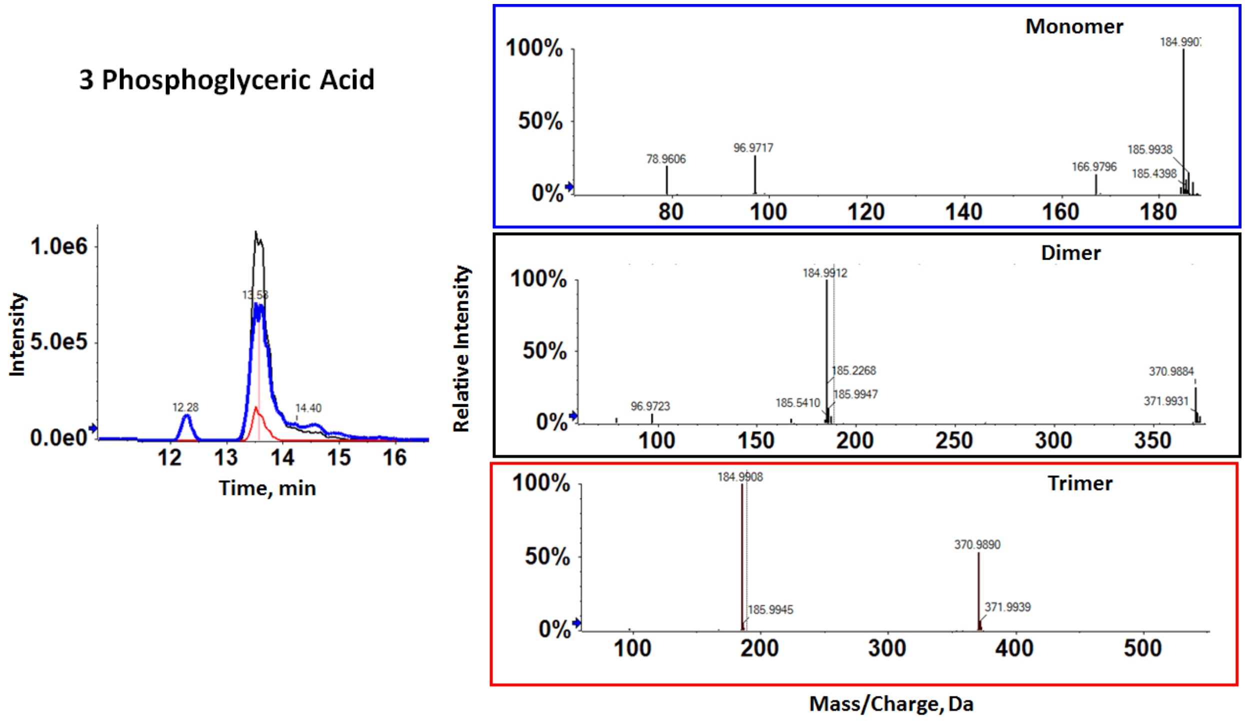

S19 Fig: Multimer ion adducts observed in an injection of a pure standard compound 3 phosphoglyceric acid. The extracted ion chromatograms (XIC) of the monomer and dimer ions is shown in the overlay plot where monomer ions, dimer ions and trimer ions are represented using blue, black and red traces respectively. The MS spectrum of the precursor ion 184.9 (monomer), 370.9 (dimer), and 556.9 (trimer) at a collision energy of -30 eV is presented with relative intensity. We observed the presence of monomer ions in the MS spectrum of the dimer and trimer ions.
