# Supplementary material for: Mass Isotopologue Distribution of dimer ion adducts of intracellular metabolites for potential applications in 13C Metabolic Flux Analysis
Source: PLoS One. 2019 Aug 21;14(8):e0220412. doi: 10.1371/journal.pone.0220412 (PMC6703694; doi:10.1371/journal.pone.0220412)
Supplement: S21 Fig — (PDF) [file pone.0220412.s023.pdf]

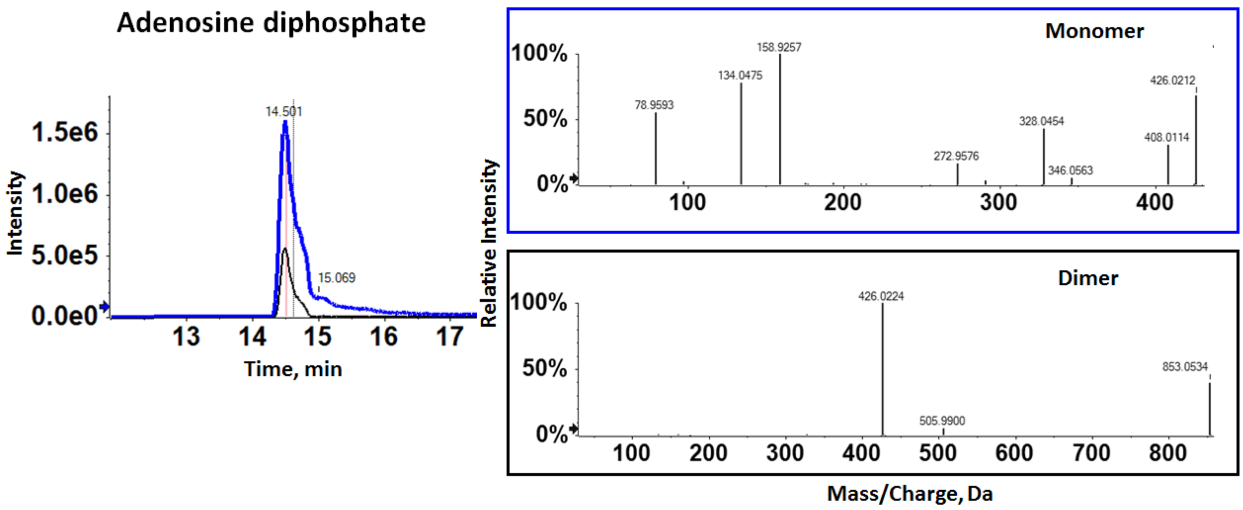

S21 Fig: Multimer ion adducts observed in an injection of a pure standard compound adenosine diphosphate. The extracted ion chromatograms (XIC) of the monomer and dimer ions is shown in the overlay plot where monomer ions and dimer ions are represented using blue and black traces respectively. The MS spectrum of the precursor ion 426.02 (monomer) and 853.06 (dimer) at a collision energy of -30 eV is presented with relative intensity. We observed the presence of monomer ions in the MS spectrum of the dimer ions.
