# Supplementary material for: Mass Isotopologue Distribution of dimer ion adducts of intracellular metabolites for potential applications in 13C Metabolic Flux Analysis
Source: PLoS One. 2019 Aug 21;14(8):e0220412. doi: 10.1371/journal.pone.0220412 (PMC6703694; doi:10.1371/journal.pone.0220412)
Supplement: S25 Fig — (PDF) [file pone.0220412.s027.pdf]

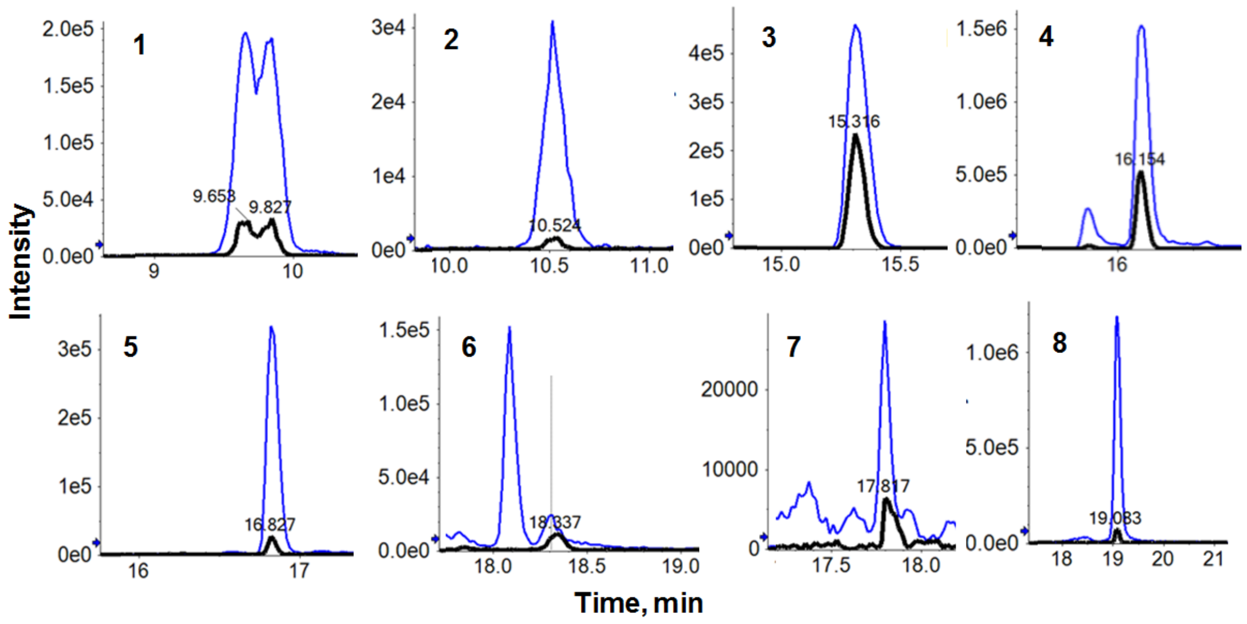

S25 Fig: Overlay plots of extracted ion chromatograms from putative monomer-dimer pairs observed in the strain *Synechococcus elongatus* PCC 11801. The corresponding retention time (RT) and the m/z values for monomer-dimer ions for each of the pairs are; compound 1, RT= 9.8 and m/z values 245.1-491.2; compound 2, RT = 10.5 and m/z values 277.08-555.17; compound 3, RT = 15.3 and m/z values 386.23-771.47; compound 4, RT = 16.2 and m/z values 254.12 – 509.24; compound 5, RT = 16.8 and m/z values 293.17-587.35; compound 6, RT = 18.3 and m/z values 353.19-635.47; compound 7, RT = 18.8 and m/z values 287.24 – 573.48 and compound 8, RT = 19.1 and m/z values 255.23 – 511.47. The extracted ion chromatogram for the monomer ion is represented in blue and that for the dimer ion is represented in black.
