# Supplementary material for: Mass Isotopologue Distribution of dimer ion adducts of intracellular metabolites for potential applications in 13C Metabolic Flux Analysis
Source: PLoS One. 2019 Aug 21;14(8):e0220412. doi: 10.1371/journal.pone.0220412 (PMC6703694; doi:10.1371/journal.pone.0220412)
Supplement: S26 Fig — (PDF) [file pone.0220412.s028.pdf]

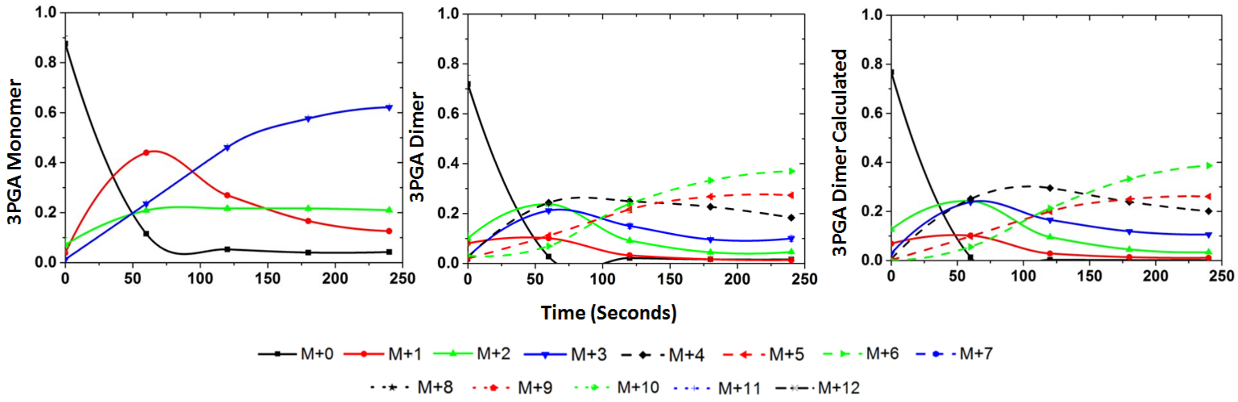


**S26 Fig: Mass isotopologue distribution ion adducts of 3PGA, quantitated from a time course measurement and the MIDs of dimer ions calculated from monomer ions.**
