# Supplementary material for: Mass Isotopologue Distribution of dimer ion adducts of intracellular metabolites for potential applications in 13C Metabolic Flux Analysis
Source: PLoS One. 2019 Aug 21;14(8):e0220412. doi: 10.1371/journal.pone.0220412 (PMC6703694; doi:10.1371/journal.pone.0220412)
Supplement: S27 Fig — (PDF) [file pone.0220412.s029.pdf]

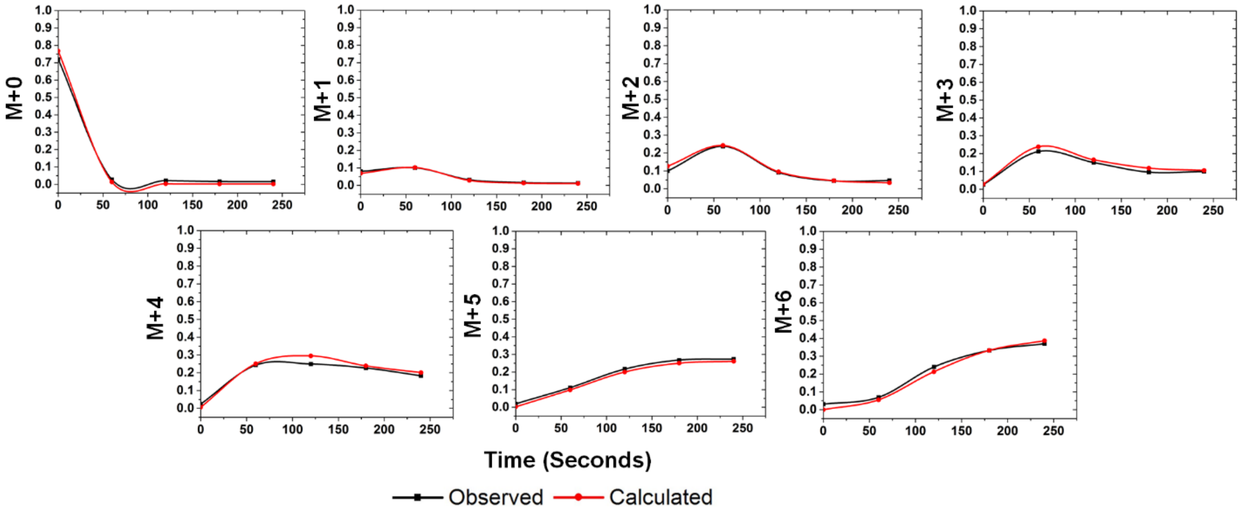

S27 Fig: Overlay plot for the mass isotopologues of dimer ion of 3PGA quantitated and calculated for dataset from *Synechococccus elongatus* PCC 11801.
